# Direct observation of ultrafast many-body electron dynamics in an ultracold Rydberg gas

**DOI:** 10.1038/ncomms13449

**Published:** 2016-11-16

**Authors:** Nobuyuki Takei, Christian Sommer, Claudiu Genes, Guido Pupillo, Haruka Goto, Kuniaki Koyasu, Hisashi Chiba, Matthias Weidemüller, Kenji Ohmori

**Affiliations:** 1 Department of Photo-Molecular Science, Institute for Molecular Science, National Institutes of Natural Sciences, Myodaiji, Okazaki 444-8585, Japan; 2 SOKENDAI (The Graduate University for Advanced Studies), Myodaiji, Okazaki 444-8585, Japan; 3 Institut für Theoretische Physik, Universität Innsbruck, Technikerstrasse 25, A-6020 Innsbruck, Austria; 4 IPCMS (UMR 7504) and ISIS (UMR 7006), University of Strasbourg and CNRS, 67000 Strasbourg, France; 5 Faculty of Engineering, Iwate University, 4-3-5 Ueda, Morioka 020-8551, Japan; 6 Physikalisches Institut, Universität Heidelberg, Im Neuenheimer Feld 226, 69120 Heidelberg, Germany; 7 Hefei National Laboratory for Physical Sciences at the Microscale and Department of Modern Physics, University of Science and Technology of China, Hefei, Anhui 230026, China; 8 CAS Center for Excellence and Synergetic Innovation Center in Quantum Information and Quantum Physics, University of Science and Technology of China, Hefei, Anhui 230026, China

## Abstract

Many-body correlations govern a variety of important quantum phenomena such as the emergence of superconductivity and magnetism. Understanding quantum many-body systems is thus one of the central goals of modern sciences. Here we demonstrate an experimental approach towards this goal by utilizing an ultracold Rydberg gas generated with a broadband picosecond laser pulse. We follow the ultrafast evolution of its electronic coherence by time-domain Ramsey interferometry with attosecond precision. The observed electronic coherence shows an ultrafast oscillation with a period of 1 femtosecond, whose phase shift on the attosecond timescale is consistent with many-body correlations among Rydberg atoms beyond mean-field approximations. This coherent and ultrafast many-body dynamics is actively controlled by tuning the orbital size and population of the Rydberg state, as well as the mean atomic distance. Our approach will offer a versatile platform to observe and manipulate non-equilibrium dynamics of quantum many-body systems on the ultrafast timescale.

## Abstract (web summary)

Studying long-range interactions in the controlled environment of trapped ultracold gases can help our understanding of fundamental many-body physics. Here the authors excite a gas of Rydberg atoms with a ps laser pulse, demonstrating behaviour consistent with many-body correlations beyond mean-field.

## Graphical abstract

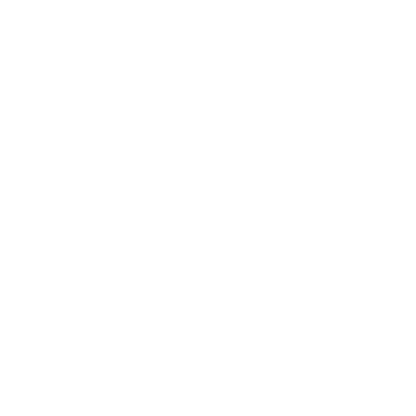

Atomic, molecular and optical physics with advanced laser technologies has recently emerged as a new platform to study and possibly simulate quantum many-body systems^1^,^2^,^3^. One of its latest developments is the study of long-range interactions among ultracold particles, in which dipolar quantum gases^4^,^5^, ion crystals^6^,^7^,^8^, polar molecules^9^,^10^,^11^ and Rydberg atoms^12^,^13^,^14^,^15^ allow for revealing the effects of many-body correlations. Rydberg atoms distinguish themselves by their large dipole moments and tunability of the strength and nature of dipolar interactions^12^,^13^.

The active electron in a Rydberg atom moves in a macroscopic orbital whose size could range from sub-micrometre to several tens of micrometres^16^. The resulting large dipole moments yield a strong interaction *U*(*r*) of either van der Waals or resonant dipolar character between a pair of Rydberg atoms separated by *r*. This interaction, which can be tuned in various ways^12^, features the emergence of atomic many-body correlations in an ultracold gas. As a prominent example, these interactions shift the energy levels of atoms encompassing a given Rydberg atom, so that additional Rydberg excitations are suppressed for *U*(*r*) larger than the excitation line width. This effect, referred to as Rydberg blockade^13^,^13^,^17^, results in spatial or temporal correlations among the atoms, as recently demonstrated in various settings^15^,^18^,^19^,^20^,^21^,^22^,^23^,^24^,^25^,^26^. Its applications include, for example, the realization of universal atomic and photonic logic gates for quantum information processing^12^,^27^,^28^,^29^,^30^,^31^. The Rydberg blockade has also turned out to be an outstanding new resource to investigate many-body problems^32^. The blockade condition determines the smallest distance between neighbouring Rydberg atoms, which is typically of the order of several micrometres. However, Rydberg excitations can also be induced at smaller interatomic distances by, for example, detuning the excitation-laser frequency from an atomic resonance^33^,^34^,^35^,^36^,^37^,^38^. In this regime, the character of the correlations changes, resulting in the facilitated formation of aggregates consisting of large number of Rydberg atoms^34^,^35^,^36^,^37^,^38^.

A complementary approach to the correlations induced by the interparticle interactions consists in studying the temporal evolution of electronic coherences of the Rydberg atoms. Using a broadband picosecond laser pulse, Rydberg excitations can be induced over a wide range of interatomic distances from <1 μm to the isolated atom limit. The number of Rydberg excitations per unit volume can thus be larger than the ones in the blockade regime by two orders of magnitude. Although picosecond and femtosecond laser pulses have been previously used to observe electronic wave packets in isolated Rydberg atoms^39^,^40^ and the dephasing due to two-body interactions^41^, here we exploit them to explore how coherent dynamics evolves in a many-body regime. The strong interactions in our ultracold Rydberg gas induce an electronic dephasing on the picosecond timescale, which is directly observed in a time-domain Ramsey interferogram oscillating with a period of ∼1 fs and phase-shifted on the attosecond timescale^42^ by the Rydberg interactions. We measure this minute phase shift and the dephasing directly, and compare them with theoretical simulations based on nearest-neighbour interactions, a mean-field model and many-body correlations to reveal effects indicating atomic correlations beyond a mean-field description. The two-body interaction energy at around 1 μm distance, which can be easily accessed in our approach, exceeds the average kinetic energy by many orders of magnitude. This regime compares favourably with previous experiments in thermal vapour cell experiments with nanosecond laser pulses^38^,^43^, where the nearest-neighbour distance is also <1 μm. In thermal cells, the Rydberg interaction energy is comparable to the average kinetic energy, so that the measurement of coherent evolution of the interaction dynamics is strongly affected by the thermal atomic motion, as will be discussed later quantitatively.

## Results

### Experimental setup

**Figure 1:**
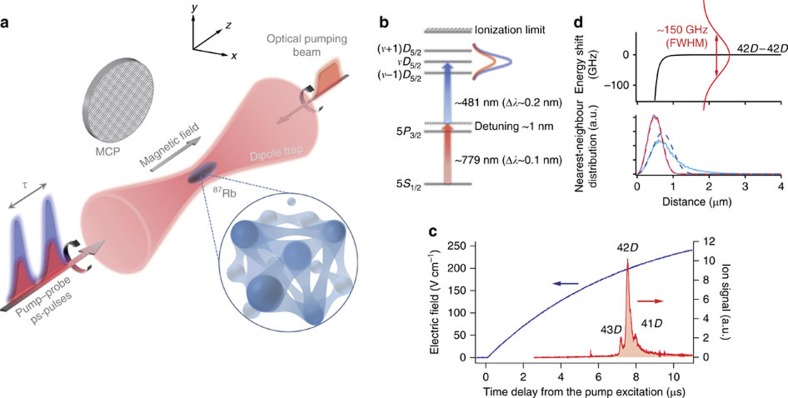
Schematic diagram of the experimental setup and Rydberg excitation. (**a**) Sketch of the experimental setup. The dipole-trap laser is turned off 2 μs before the irradiation of the picosecond pulses, to avoid 2+1 multiphoton ionization induced by a combination of the picosecond pulses and the trapping laser beam. (**b**) Two-photon pump (probe) excitation of the Rb atom to its Rydberg states. (**c**) The Rydberg states can be resolved by field ionization with a slowly ramped electric field (see Methods section ‘Rydberg excitation and detection'). Here, the Rydberg population and estimated peak atom density were 1.2±0.1% and ∼4 × 10^10^ cm^−3^, respectively (see Methods sections ‘Rydberg excitation and detection' and ‘Estimation of the atom density' for these population and density estimations). The small peak around 5.6 μs could be assigned to free ions generated by population redistribution^47^ and/or direct multiphoton ionization. (**d**) Sketch of the two-body interaction and pulse excitation accompanied by a plot of the nearest-neighbour distribution of Rydberg atoms for the peak atom density of *n*=1.3 × 10^12^ cm^−3^ (pink solid and red dashed traces) and the averaged density over the whole atoms (light-blue solid and dark-blue dashed traces) estimated for the present ensemble of the Rb atoms (see Methods section ‘Estimation of the atom density'). Here, the pink and light-blue solid traces are obtained by a Monte-Carlo simulation, whereas the red and dark-blue dashed traces show analytical results for homogeneous distributions. The difference between the light-blue and dark-blue average density traces results from the difference between their Gaussian and homogeneous density distributions, respectively, over the whole ensemble. At the peak atom density, the average nearest-neighbour distance is given by 0.5 μm, whereas for the averaged density we obtain 0.87 μm.

Figure 1a shows the schematics of our experimental setup. A cold ensemble of ^87^Rb atoms is prepared in an optical dipole trap with temperature and highest peak atom-density estimated to be ∼70 μK and ∼1.3 × 10^12^ cm^−3^, respectively (see Methods sections ‘Estimation of the temperature' and ‘Estimation of the atom density' for these temperature and density estimations). On the timescales of interest, atomic motion can be ignored in the frozen gas regime^44^,^45^. The atoms are optically pumped to the hyperfine state *F*=2, *m*_F_=+2 of the ground state 5*S*_1/2_ and excited to Rydberg states via a two-photon transition using broadband picosecond laser pulses with their centre wavelengths tuned to ∼779 and ∼481 nm (Fig. 1b), hereafter referred to as the infrared and blue pulses, respectively. The dipole-trap laser is turned off 2 μs before the irradiation of the infrared and blue pulses to avoid 2+1 multiphoton ionization induced by a combination of the infrared and blue pulses with the trapping laser light. The infrared and blue pulses, and the optical pumping beam are circularly polarized in the same direction with respect to the magnetic field, suppressing excitations to the *S* Rydberg states, so that the state *vD*_5/2_, *m*_J_=+5/2 is mostly populated, where *v* is a principal quantum number. The population of the Rydberg state is measured by field ionization^16^. Details of the field ionization are described in Methods section ‘Rydberg excitation and detection'. The maximum population of the *vD* states in the present experiment is not >5%, to suppress photoionization by the picosecond laser pulses (see Supplementary Note 1 for the effects of ions). More details on the atom preparation, the estimation of the atom density and the temperature, as well as the Rydberg excitation are described in Methods and Supplementary Note 2.

The bandwidth of our Rydberg excitation is determined from a field-ionization spectrum exemplified in Fig. 1c (see Supplementary Note 3), in which the excitation is tuned to the 42*D*_5/2_ state, which is the main target state of the current experiment. The field-ionization voltage is ramped up slowly enough on the 5 μs timescale to resolve neighbouring Rydberg levels in such field-ionization spectra. The bandwidth of the excitation is ∼150 GHz (full width at half maximum, FWHM) and is much larger than those of the continuous-wave, nanosecond, and sub-nanosecond pulsed lasers employed in previous ultracold Rydberg experiments^12^,^13^,^41^,^46^,^47^. The field-ionization spectrum indicates that the bandwidth of the Rydberg excitation is larger than the energy separation of neighbouring Rydberg states and is accordingly wide enough to remove the Rydberg blockade. As schematically shown in the upper panel of Fig. 1d, our picosecond laser pulses can excite a pair of Rb atoms simultaneously to the Rydberg states *v*=42 even at interatomic distances shorter than 1 μm.

### Observation of many-body electron dynamics

The interaction among the Rydberg atoms is observed by time-domain Ramsey interferometry with a pair of the Rydberg excitations, hereafter referred to as the ‘pump' and ‘probe' excitations, whose delay was stabilized on the attosecond timescale with our homemade optical interferometer^48^ (see Methods section ‘Time-domain Ramsey interferometry'). By scanning the delay time *τ* between the pump and probe excitations on the attosecond timescale, we measure the Ramsey oscillation of the population integrated over all Rydberg states, which remains after the probe excitation, by the field ionization. The field-ionization voltage in these Ramsey measurements is ramped up rapidly enough on the 100 ns timescale to avoid subsequent decay processes of the Rydberg states. For the excitation tuned to the 42*D*_5/2_ state as shown in Fig. 1c, a single Rydberg state |*v*〉 with *v*=42 is predominantly populated. The Rydberg population thus measured oscillates as a function of *τ* with a frequency close to the transition frequency between the 5*S* and Rydberg states^42^. This is in contrast to the standard Ramsey interferometry, in which the signal oscillates with a frequency close to the detuning frequency of the excitation laser from the atomic transition^22^,^23^. That is, in the absence of interactions, the population in the Rydberg state *P*_*v*_(*τ*) is given by


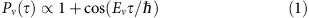


and oscillates with the frequency *E*_*v*_/*h*, where *E*_*v*_ is the energy of the Rydberg state |*v*〉 measured from the ground state 5*S* (see refs 42, 49 and Supplementary Note 2) and *ℏ* is the Planck constant *h* divided by 2π. This oscillation is identical to the temporal oscillation of the Rydberg state |*v*〉, except that the real-time *t* is replaced by the pump-probe delay *τ*. Therefore, the Ramsey oscillation for *v*=42 corresponds essentially to the temporal oscillation of the Rydberg wave function |*v*=42〉 and to the recurrence motion of an electronic wave packet, which is composed of the 5*S* and Rydberg state *v*=42 superposed coherently by the excitation pulses. Here we investigate how the Rydberg interactions affect this coherent electron dynamics.

In a simplified mean-field approach, the Rydberg interactions change only the energy of the Rydberg state |*v*〉 and accordingly the oscillation period of *P*_*v*_(*τ*). This results in a phase-shift of the Ramsey oscillation accumulated in the delay time *τ*. In addition, as the atoms are randomly distributed in the ensemble, their energy levels *E*_*v*_ are shifted randomly by the interactions, making the periods of their Ramsey oscillations different from each other. Therefore, the measured signal is the superposition of many oscillations with different periods, so that its contrast is expected to decay as a function of *τ* due to Rydberg interactions.

**Figure 2:**
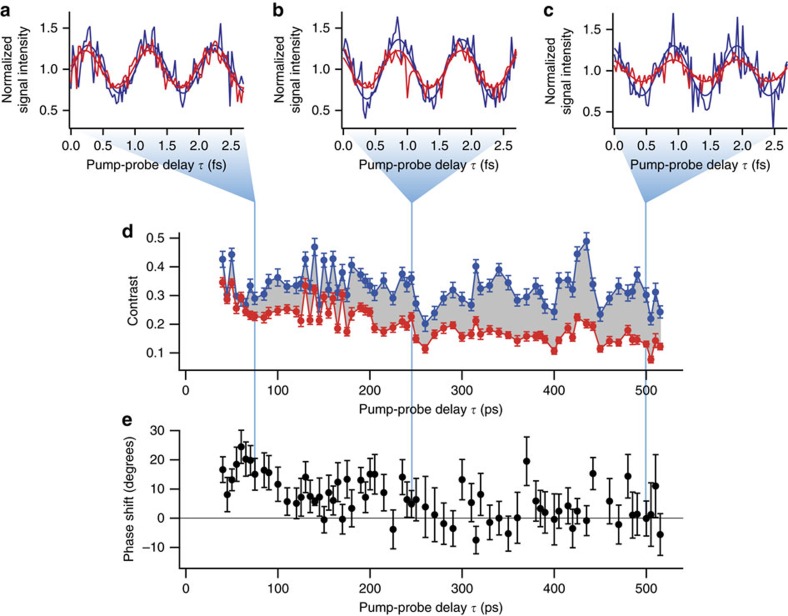
Ramsey oscillation for the 42*D* state. (**a**–**c**) The field ionization signals for the higher-density (red traces) and the lower-density (blue traces) ensembles are plotted as functions of the pump-probe delay *τ* scanned over a range of ∼3 fs around 75, 245 and 500 ps, showing clear oscillations. The signal intensities are normalized by the mean value of the sinusoidal function fitted to each oscillation. The origin of the pump-probe delay *τ*=0 is arbitrary and is taken to be the left edge of each figure. (**d**) The contrasts of these oscillations are plotted as functions of *τ.* The oscillatory structures as functions of *τ* could be partly attributable to the recurrence motion of a wave packet composed of the 42*D* and its neighbouring Rydberg states (see the field-ionization spectrum shown in Fig. 1c). The shortest period of this recurrence motion is evaluated from their level spacing to be ∼10 ps, which is not resolved in these plots. More details on the oscillatory structures are described in Supplementary Note 4. The contrast decays clearly as a function of *τ* for the higher-density ensemble, whereas for the lower-density one a decay is not visible. (**e**) The phase shift of the higher-density ensemble from the lower-density one is plotted as a function of *τ*. The oscillatory structure as a function of *τ* is partly attributable to the wave-packet motion. The error bars represent the s.d.

Figure 2a–c show examples of the Ramsey oscillations for the 42*D*_5/2_ state with a population of 3.3±0.1%. As mentioned above, these oscillations correspond to the ultrafast recurrence motion of the electronic wave packet with a period of ∼1 fs. For each oscillation, we measured the field-ionization signals of two atomic ensembles with different peak densities estimated to be ∼1.3 × 10^12^ and ∼4 × 10^10^ cm^−3^ alternately to suppress systematic uncertainties, scanning *τ* in steps of ∼30 as (see Methods section ‘Estimation of the atom density' for these density estimations). We obtained the contrasts and phases of the measured oscillations by sinusoidal fitting, as shown in Fig. 2a–c (see Methods section ‘Time-domain Ramsey interferometry'). Figure 2d shows that the contrast is approximately constant for *τ* up to ∼500 ps for the lower-density ensemble (blue-circle data points), indicating that the effects of the interactions are negligibly small. This result is consistent with the interaction strength estimated from the present atom density and the two-atom potential curve presented in Supplementary Fig. 1. Hereafter, we take these contrasts and phases measured in the lower-density ensemble as references to be compared with those measured in the higher-density ensemble. The oscillatory structures on the ∼10 ps timescale at *τ*∼130–170 ps seen in Fig. 2d could be partly due to the recurrence motion of a wave-packet composed of the Rydberg state *v*=42 and the traces of its neighbouring states seen in Fig. 1c (see Supplementary Note 4 for details).

Figure 2d shows that the contrast decays as a function of *τ* for the higher-density ensemble (red-circle data points). The phase shift of the higher-density ensemble from the lower-density one also changes as a function of *τ* as seen in Fig. 2e. The offset of this phase shift at *τ*=0 is essentially due to the difference between AC-Stark shifts of the atomic levels in the higher- and lower-density ensembles. Slight differences between the sizes, shapes and positions of the higher- and lower-density ensembles could lead to their different AC-Stark shifts (see Supplementary Note 2 for more details on the origin of this zero-delay offset of the phase shift).

**Figure 3:**
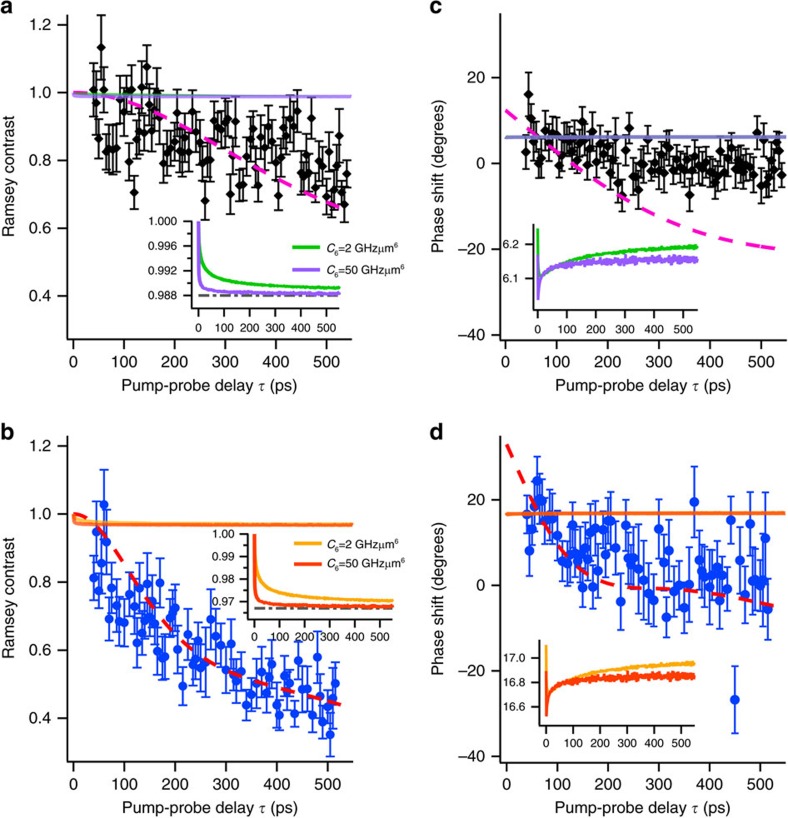
Measured Ramsey contrasts and phase shifts compared with those expected for nearest-neighbour interactions and mean-field approximations. The black diamond-shaped and blue circle data points show the measured Ramsey contrasts (**a** and **b**) and the phase shifts (**c** and **d**) for the population *p*_e_∼1.2% (**a** and **c**) and ∼3.3% (**b** and **d**) of the 42*D*_5/2_ state, respectively. In **a** and **b**, the measured Ramsey contrasts are compared with the simulated ones for nearest-neighbour interactions with a *C*_6_ coefficient of 2 GHz μm^6^ (green and yellow solid lines) and 50 GHz μm^6^ (purple and orange solid lines), respectively. Results of the mean-field simulations are presented by magenta and red dashed lines in **a** and **b**, respectively. Similarly, the measured and simulated phase shifts are compared in **c** and **d**. The zero-delay offset of the simulated phase shift is arbitrary and adjusted so that the average of the first ten data points is equal to the calculated phase shift averaged over the delay window for those ten data points. In the insets in **a** and **b**, the Ramsey contrasts simulated for the nearest-neighbour interactions (solid lines) are vertically magnified to show their convergence as the *C*_6_ value is increased. Grey dot-and-dash lines show their population-dependent thresholds given by 1−*p*_e_. In the insets in **c** and **d**, the phase shifts simulated for the nearest-neighbour interactions (solid lines) are magnified vertically. The peak atom density is set to ∼1.3 × 10^12^ cm^−3^ in these simulations. The error bars represent the s.d.

Figure 3 shows the results of measurements of the contrast ratio between the two different densities given above, hereafter referred to as ‘Ramsey contrast', and the phase shift for the 42*D*_5/2_ state. These results are plotted as functions of *τ* for the two different Rydberg populations *p*_*e*_ of 1.2±0.1% and 3.3±0.1%, showing that the Ramsey contrast decays, and the phase shift is accumulated as a function of *τ*.

**Figure 4:**
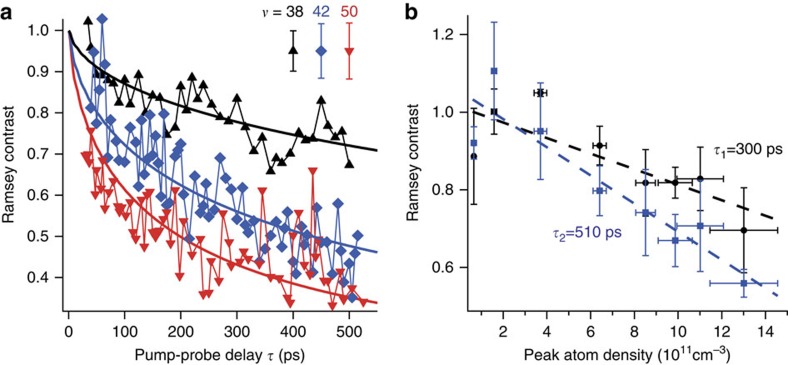
The principal quantum number and atom density dependences of the Ramsey contrast. (**a**) Measured Ramsey contrasts are plotted as functions of *τ* for three different Rydberg levels *v*=38, 42 and 50. The estimated populations *p*_e_ and peak atom densities are *p*_e_∼3.2% and ∼1.2 × 10^12^ cm^−3^ for *v*=38, *p*_e_∼3.3% and ∼1.3 × 10^12^ cm^−3^ for *v*=42, and *p*_e_∼3.1% and ∼1.2 × 10^12^ cm^−3^ for *v*=50, respectively (see Methods sections ‘Estimation of the atom density' and ‘Rydberg excitation and detection' for these population and density estimations). The simulations indicated by the black, blue and red solid lines yield adjusting parameters to be *C*_6_=8, 34 and 103 GHz μm^6^ for *v*=38, 42 and 50, respectively. The interaction strength in these simulations is limited below 75 GHz, which is the half width half maximum of the pump excitation, and the peak atom density is set to the estimated density for each Rydberg level in these simulations. It should be noted that several Rydberg states are excited for *v*=50 (see Supplementary Fig. 4). However, we consider an excitation to a single Rydberg state, to perform the simulations for all of the three Rydberg levels. Each error bar in the inset represents the average over the error bars (the s.d.) of all data points for each Rydberg level. (**b**) Ramsey contrast is measured as a function of the peak atom density at two different pump-probe delays *τ*=300 and 510 ps for *v*=42 with its population being ∼3.5%. The vertical error bars represent the s.d. and the horizontal error bars arise from the density calibration (see Supplementary Note 5 for the estimation of the atom densities plotted in the abscissa).

It is evident from Fig. 3 that the contrast decay and the phase shift are enhanced when the Rydberg population is increased from ∼1.2% to ∼3.3%. The strength of the interactions is tuned by varying the Rydberg quantum number *v* and the atom density. Figure 4a shows Ramsey contrasts as functions of *τ* for three different Rydberg levels *v*=38, 42 and 50. It is seen from this figure that the contrast decay is accelerated by increasing the principal quantum number *v* of the Rydberg level. The dependence of the Ramsey contrast decay on the atom density is shown in Fig. 4b, in which the contrast decay is accelerated by increasing the atom density (see Supplementary Note 5 for the estimation of the atom densities plotted in the abscissa of this figure). From these combined measurements as functions of the Rydberg population, principal quantum number and atom density, we conclude that the observed contrast decay and the phase shift are induced by Rydberg interactions.

The origin of the observed behaviour of the contrast decay and the phase shift is further investigated in Fig. 3. Here we compare the experimental data with the Ramsey contrast decays and the phase shifts calculated for nearest-neighbour interactions without considering interactions among three or more Rydberg atoms (solid lines). The zero-delay offset of the calculated phase shift is arbitrary and adjusted so that the average of the first ten data points is equal to the calculated phase shift averaged over the delay window for those ten data points. Results for nearest-neighbour interactions are obtained by simple numerical calculations following those performed in previous Ramsey studies^23^,^41^, in which the time-domain Ramsey oscillations modulated by nearest-neighbour interactions are averaged over the distribution of nearest-neighbour distances (see Supplementary Note 6 for details). Here, the Ramsey oscillation is obtained by solving a Schrödinger equation with a Hamiltonian for two interacting atoms. We assume a van der Waals interaction of the form *U*(*r*)=−*C*_6_/*r*^6^ with the coefficient *C*_6_ being an adjusting parameter. It is seen in the insets of Fig. 3a,b that the calculated Ramsey contrast decay becomes slightly faster as the *C*_6_ value is increased, but does not grow beyond the decay thresholds 0.988 and 0.967 determined by the Rydberg population *p*_e_∼1.2% and 3.3%, respectively (for details on these thresholds, see Supplementary Note 6). Similarly, the calculated phase shift is almost constant as the *C*_6_ value is increased, as shown in Fig. 3c,d. These features are not altered by changing the character of the interaction such as van der Waals and dipole–dipole (see Supplementary Note 6 for details). The contrast decays and phase shifts observed experimentally are thus clearly larger than expected for nearest-neighbour interactions. It is therefore concluded that our experimental observation of the Ramsey contrast decay and phase shift on the attosecond timescale demonstrates interactions among more than two Rydberg atoms, and the effect of those interactions on the electron dynamics can be actively controlled by tuning the population and principal quantum number of the Rydberg level, as well as the atom density.

### Test of a mean-field model

To model these observations, we first apply a mean-field model (see Supplementary Note 7 for its details). As in the calculations with nearest-neighbour interactions above, we assume a van der Waals interaction of the form *U*(*r*)=−*C*_6_/*r*^6^ with the coefficient *C*_6_ being the only fitting parameter, which is optimized to reproduce the measured Ramsey contrast in Fig. 3b by a least-squares fitting. The outline of the least-squares fitting is given in Supplementary Note 8. The results of the mean-field simulations are plotted in Fig. 3 (dashed lines). Figure 3a,b show that reasonable agreements can be found for the Ramsey contrast between the measurements and the mean-field simulations with *C*_6_=1.9 GHz μm^6^. This value for *C*_6_ yields the phase shifts simulated by the mean-field model in Fig. 3c,d, where the zero-delay offset is adjusted in the same way as in the calculations with the nearest-neighbour interactions above. Figure 3c shows that the mean-field simulation yields a phase-shift larger than the measured one by a factor of ∼4, failing to reproduce our observations. This discrepancy between measured and simulated phase shifts is not improved by assuming a dipole–dipole interaction, a hybrid form of a dipole–dipole and a van der Waals interaction or by introducing anisotropic interactions (see Supplementary Figs 2 and 3). The closer agreement between the measured and simulated phase shifts for *p*_e_∼3.3% in Fig. 3d than for *p*_e_∼1.2% in Fig. 3c is understood as follows. The Gaussian atom-density distribution of our experimental setup leads to the saturation of the phase shift at longer pump-probe delays. This is because the rapid decrease of the atom density in the Gaussian tails results in the suppression of the contribution to the phase shift from atoms distant from the centre, and therefore the phase shift does not grow afterwards. This saturation is reached both by the measured and simulated phase shifts for *p*_e_∼3.3% within our measurement time 500 ps (unlike the case with *p*_e_∼1.2% and therefore weaker interactions), giving their closer agreement for *p*_e_∼3.3% than for *p*_e_∼1.2%. This saturation effect is explained more quantitatively in Supplementary Note 9.

### Beyond mean-field analysis

Next, we apply an exactly solvable theory model^50^,^51^,^52^,^53^,^54^ to the observations. Details of this model are presented in ref. 54. Briefly, we represent each atom as a two-level system, which is a pseudo-spin system, consisting of a ground state |g〉 (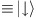) and an excited Rydberg state |e〉 (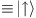) with energies *E*_g_ and *E*_e_, respectively. The experiment consists of four stages: (i) the pump excitation, (ii) an evolution with an *N*-atom Hamiltonian *H*, (iii) the probe excitation and (iv) the population measurement by field ionization. The *N*-atom Hamiltonian in stage (ii) includes the atomic energies and the interactions *U*(*r*_*jk*_) between atoms in the Rydberg states as follows:


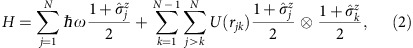


with 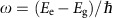 the atomic-resonance frequency and 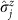 the Pauli matrix, which is used to represent the internal states of the pseudo-spin at position *j* (see Methods section ‘Outline of the exactly solvable model simulation' for more details)^50^,^51^,^52^,^53^,^54^.

**Figure 5:**
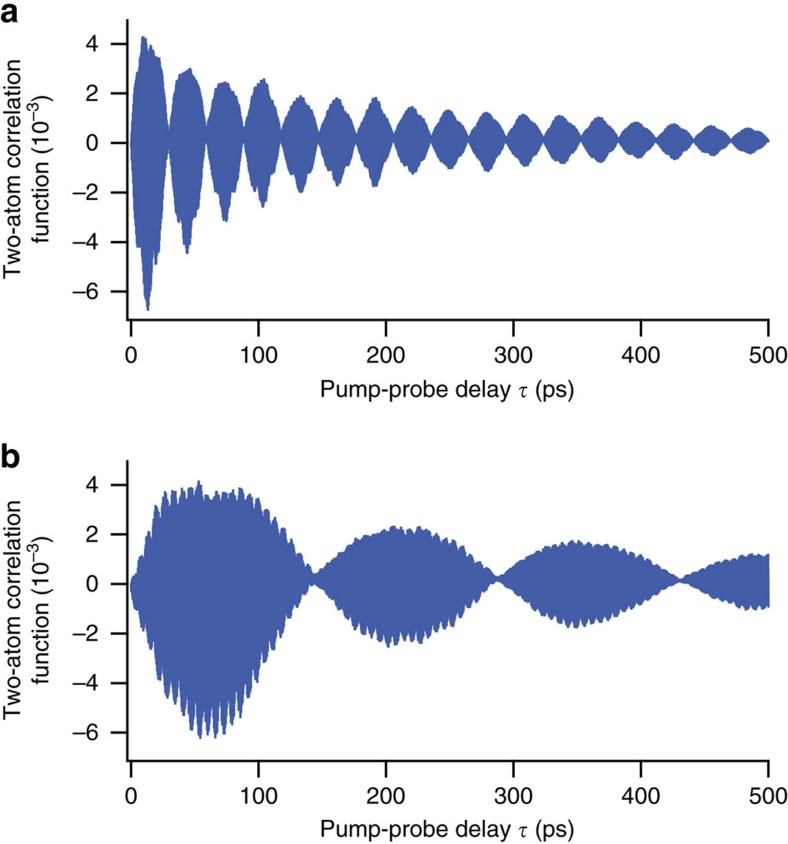
Two-atom correlation simulated by the exact model. The two-particle correlation function is calculated for two atoms in the Gaussian atom density distribution of the present Ramsey measurements for the Rydberg level *v*=42. The one atom is located at the centre of the distribution, and the distance to the other atom is set to 1 μm (**a**) and 1.3 μm (**b**), respectively. The *C*_6_ coefficient, the Rydberg population and the peak atom density are set to 34 GHz μm^6^, ∼3.3% and ∼1.3 × 10^12^ cm^−3^, respectively. The interaction strength is limited below 75 GHz, which is the half width half maximum of the pump excitation. The coherent superposition of different clusters of interactions leads to dephasing and therefore the global decay.

An exact solution for the time evolution with a Hamiltonian of the form of equation (2) has recently been presented in refs 50, 51, 52, 53, 54 (see also similar numerical approaches in refs 55, 56). This allows for deriving an expression for the exact time evolution of the Ramsey signal *P*(*τ*) for any strength of interactions^50^,^51^,^52^,^53^,^54^. For any given atom *j* interacting with *N*–1 neighbouring Rydberg atoms, one obtains


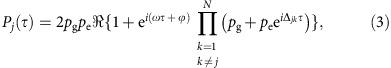


where *p*_g_ and *p*_e_ are the ground- and Rydberg-state populations, respectively, produced by the initial pump excitation, 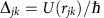 describes a frequency shift induced by the interaction between atoms *j* and *k*, and *ϕ* is the phase offset arising from the AC-Stark shifts during the picosecond pulse excitations (see Supplementary Note 2). It is seen from this equation that this model considers different clusters of interactions superposed coherently instead of averaged as performed in our preceding mean-field analysis. This coherent superposition leads to correlations among different atoms, as exemplified for a two-atom correlation in Fig. 5 (ref. 54).

Further analytical progress is possible by using an approximation, hereafter referred to as a ‘continuum approximation', in which a continuum function *n*(**r**) is considered for the density distribution of Rydberg atoms (see Methods section ‘Continuum approximation' for details). Briefly, we assume the density distribution to be homogeneous in a small volume around a particular position **r** (ref. 54). This approximation leads to the following expression for the Ramsey signal averaged over the whole atomic ensemble


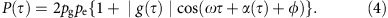


The contrast decay |*g*(*τ*)| and phase shift *α*(*τ*) are then obtained from 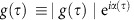, which, in the case of an isotropic van der Waals interaction and for 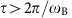, is given by


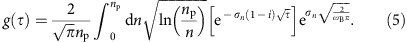


Here, *ω*_B_=2*π* × 75 GHz is the half width half maximum of the pump excitation, *n*_p_ is the peak atom density, the coefficient 
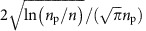
 results from the Gaussian atomic density distribution in the experiment and 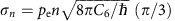 is the decay constant^54^. Equation (5) predicts that the Ramsey contrast decays approximately as a stretched exponential 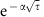 with a square-root dependence on the pump-probe delay *τ*. This square-root dependence is characteristic of the van der Waals interaction^54^. The decay constant reads 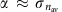, where 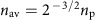 is the average density for a Gaussian distribution.

**Figure 6:**
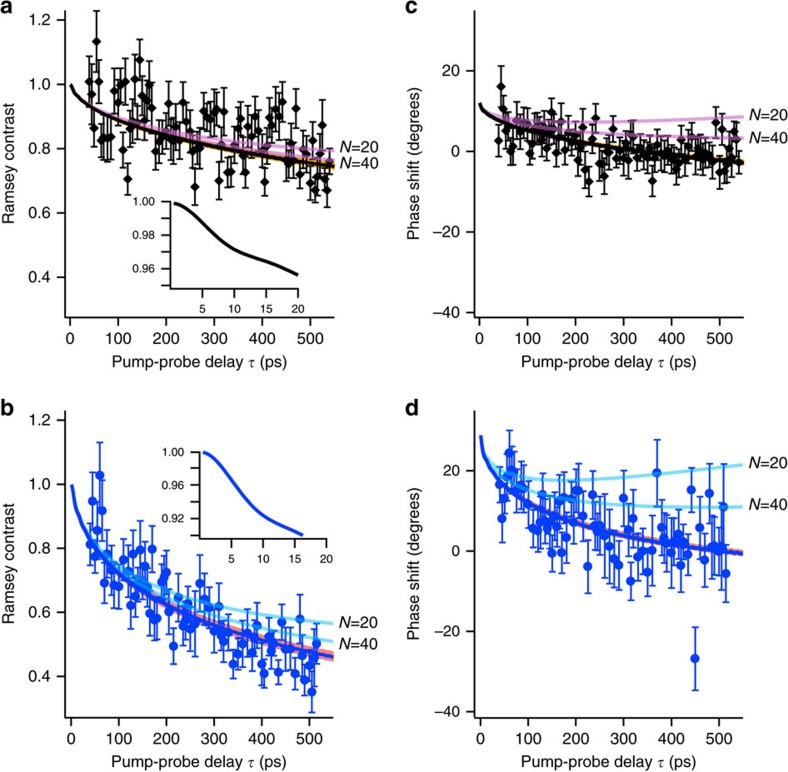
Measured Ramsey contrasts and phase shifts compared with the numerical ones based on the analytical continuum approximation equation (4). The black diamond-shaped and blue circle data points show the measured Ramsey contrasts (**a** and **b**) and the phase shifts (**c** and **d**) for the population of the 42*D*_5/2_ state being ∼1.2% (**a** and **c**) and ∼3.3% (**b** and **d**), respectively. In **a** and **b**, the measured Ramsey contrasts are compared with the numerical ones based on the analytical continuum approximation for the average number of Rydberg atoms *N*=20 and *N*=40 (purple and blue semi-transparent lines, respectively), as well as for the limit 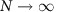 (black and blue solid lines). The curves for the exact solution agree well with the ones for 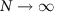 in the continuum approximation, so that they are indistinguishable on the present scale of the figure. The orange and pink shaded areas correspond to 2 s.d. of the *C*_6_ coefficient obtained in the least-squares fitting. In the insets in **a** and **b**, the analytical results in the limit are magnified to show their early quadratic decays at small pump-probe delays originating from the limited bandwidth given by 75 GHz, which is the half width half maximum of the pump excitation. Similarly, the measured and numerical phase-shifts are compared in **c** and **d**. The peak atom density is set to ∼1.3 × 10^12^ cm^−3^ in these simulations. The error bars represent the s.d.

**Figure 7:**
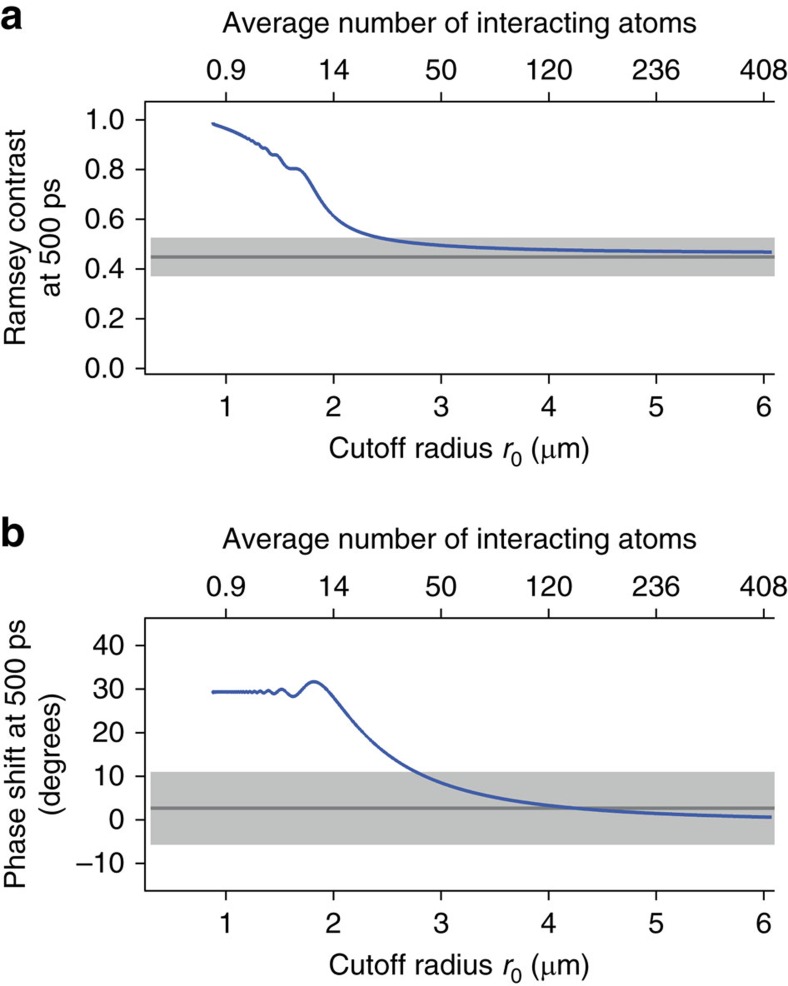
Convergence of the simulated Ramsey contrast and phase shift as functions of an average number of interacting atoms. The Ramsey contrast (**a**) and phase shift (**b**) at *τ*=500 ps are simulated by the theory model with the continuum approximation and are plotted as functions of the cutoff radius *r*_0_ (the lower abscissa) and of an average number of interacting atoms within the volume 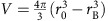 (the higher abscissa), where *r*_B_ is the blockade radius (see Methods section ‘Continuum approximation' for more details of *r*_0_ and *r*_B_). The population of the 42*D*_5/2_ is set to ∼3.3% in these simulations. The interaction strength is limited below 75 GHz, which is the half width half maximum of the pump excitation, and the peak atom density is set to ∼1.3 × 10^12^ cm^−3^ in these simulations. The results with the van der Waals interaction are displayed by the blue solid lines. The dark-grey solid lines represent the measured Ramsey contrast and the phase shift, each of which is the average over eight points around *τ*=500 ps in Fig. 6b,d. The light-grey shaded area represents 1 s.d. of the average over those eight measured values. Similar results with a dipole–dipole interaction and a hybrid form of a dipole–dipole and a van der Waals interaction are shown in Supplementary Fig. 5.

Figure 6 shows comparisons between (i) the experimental data for the contrast decay and phase shift as functions of *τ* for the 42*D*_5/2_ state and (ii) their numerical results based on the analytical continuum approximation equation (4) (solid lines). The curves for the exact numerical results agree well with the analytical ones for 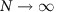, so that they are indistinguishable on the present scale of the figure. The figure demonstrates that both the exact and analytical results obtained for *C*_6_=34 GHz μm^6^ agree well with the measured Ramsey contrasts and phase shifts for both of the Rydberg populations *p*_e_∼1.2% and 3.3% used in the measurement. Figure 6 also shows numerical results with the continuum approximation for the Ramsey contrasts and phase shifts obtained for a finite average number of interacting Rydberg atoms *N*=20 and 40 (semi-transparent lines; see Methods section ‘Continuum approximation'). The agreement between the numerical and experimental results improves monotonically with increasing *N*. Figure 7 shows the Ramsey contrast and phase shift measured (the dark-grey solid lines accompanied by light-grey shaded areas) and simulated with the continuum approximation (the blue solid lines) at the pump-probe delay *τ*∼500 ps. It is seen from Fig. 7b that the blue solid line crosses the upper boundary of the light-grey shaded area, which is a confidence interval of the measured value, at an average number of interacting atoms around 40, showing that more than ∼40 atoms correlated are necessary to reproduce the measured phase shift.

In the theory model above, the *C*_6_ coefficient of the van der Waals interaction serves as the only fitting parameter for the Ramsey contrast in Fig. 6b. The *C*_6_ value thus obtained is used for calculating the contrast decay in Fig. 6a and the phase shifts in Fig. 6c,d. Then, the zero-delay offset of the phase shift serves as the only adjustable parameter for the phase shift in each of Fig. 6c,d. For distances shorter than the average interatomic separation, the bandwidth of our Rydberg excitation with the picosecond infrared and blue pulses covers multiple adiabatic interaction potentials that can hybridize with the one correlating asymptotically to the 42*D*+42*D* limit (see Supplementary Fig. 1). Although in principle this could affect the contrast decay, the present model based on a single effective potential captures the observed dynamics both for the Ramsey contrast and the phase shift (see Supplementary Note 10 for the effective treatment of the interaction potentials).

We note that the predicted decay constant for the Ramsey contrast for *p*_e_∼3.3% is approximately given by 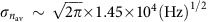. The pump-probe delay to reach e^−1^(∼0.37) of the initial contrast is thus given by 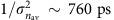, which agrees well with our measurement time of 500 ps giving the contrast reduction to 45%.

We have further employed *g*(*τ*) in equation (5) to reproduce the measured Ramsey contrasts shown in Fig. 4a for three Rydberg levels *v*=38, 42 and 50, finding good agreement. The phase shifts for these levels have also been measured and analysed in Supplementary Note 5. It should be noted that several Rydberg states are excited for *v*=50 (see Supplementary Fig. 4). However, the simulation with a single Rydberg state gives good agreement with the experimental results as shown by the solid lines in Fig. 4a.

We have also performed additional calculations of the measured Ramsey contrasts and phase shifts using dipole–dipole interactions, a hybrid form of a dipole–dipole and a van der Waals interaction, and an anisotropic van der Waals interaction. Although all results are in qualitative agreement with the experimental data, we find that the results for pure van der Waals interactions presented above reproduce the experimental data well with only a single fitting parameter. The results of these additional calculations are given in Supplementary Note 10.

### Origin of the failure of a mean-field model

The overestimation of the phase shift by the mean-field approximation in Fig. 3c is intuitively understood as follows. In our actual measurement, the Ramsey contrast of each atom decays due to many different frequencies corresponding to different clusters of interactions superposed coherently, as is expected from equation (3) of the exact theory model. In the mean-field model, however, the Ramsey oscillation of each atom has its own mean frequency, interacting with ‘the other atoms' as a whole, so that its contrast does not decay, but the decay arises only from an ensemble average of Ramsey oscillations of many atoms phase shifted from each other. Accordingly, those phase shifts need to be overestimated, as seen in Fig. 3c, to reproduce the measured contrast decay.

**Figure 8:**
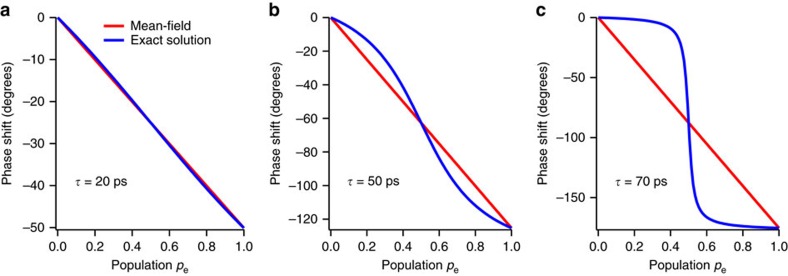
Comparison between the phase shifts obtained by the mean-field and exact calculations. In **a**–**c**, the phase shifts obtained by the mean-field (red solid lines) and exact (blue solid lines) calculations are plotted as functions of the Rydberg population *p*_e_ at the pump-probe delays *τ*=20, 50 and 70 ps, respectively. The frequency shifts induced by the interactions are set to *U*=6.96 GHz, common to all the pairs of atoms. This value corresponds to the energy shift with *C*_6_=34 GHz μm^6^ at the average internuclear distance in a Gaussian ensemble with its peak density 1.3 × 10^12^ cm^−3^.

Figure 8 shows a comparison between the phase shifts calculated by the mean field and exact models as functions of the Rydberg population *p*_e_ at three different pump-probe delays *τ*=20, 50 and 70 ps. It is seen from this figure that the mean-field phase shift is larger than the exact one for *p*_e_<0.5. It is also seen from this figure that this difference becomes larger as the effective number of interacting atoms becomes larger at longer pump-probe delays. The mean-field model thus overestimates the phase shift for the present *p*_e_∼0.01 and 0.03 (<0.5), and *τ*∼50–500 ps, as is intuitively understood as described in the preceding paragraph.

## Discussion

The experiments presented here bear similarities with recent investigations on coherent spin-exchange dynamics with rotational states of polar molecules trapped in an optical lattice^9^,^10^. In these experiments, the effect of single-particle decoherence, which proceeds faster than the interaction timescale (∼10 ms), has successfully been circumvented by applying spin echo techniques. In our experiment, on the other hand, the interaction timescale ∼1 ns is about three orders of magnitude shorter than the timescale of single-particle decoherence, which is induced mainly by Doppler broadening and is ∼1 μs at the temperature ∼100 μK estimated for our Rydberg gas (see Methods section ‘Estimation of the temperature'). The combination of ultrafast and ultracold approaches thus provides an effective pathway for isolating the observation and control of coherent dynamics of a system from its single-particle decoherence processes.

Experiments similar to the ones demonstrated here could in principle be implemented in thermal cells^38^,^43^ if one can compensate for the relevant Doppler shift, which causes a phase shift comparable to the interaction-induced phase shift, and can also compensate the effects of atomic motions. In the thermal cell at a room temperature, the atoms are not frozen, but move with kinetic energies at that temperature, so that the motion would lead to additional phase shifts on the order of 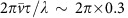 under the conditions (*λ*∼297 nm, *τ*∼500 ps, 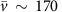 ms ^−1^), where *λ* is the wavelength that corresponds to the energy difference between the ground and Rydberg states. In our current ultracold measurements, on the other hand, the atoms move only 40 pm on average during the same duration, and this distance is shorter than *λ* by four orders of magnitude. This distance is also shorter than the average nearest-neighbour distance in our Rydberg gas by four orders of magnitude (see Methods section ‘Estimation of the atom density'). Our Rydberg gas is therefore safely regarded as a frozen gas, so that the effects of the atomic motions and collisions on the measurements of coherent dynamics are negligible.

In our present study, a combination of the contrast and phase measurements serves as a useful tool to observe the effects of many-body correlations. The correlations would be further verified by additional measurements of variance in Ramsey signals, as is performed in refs 8, 57, to observe similar many-body correlation effects. Combining a microscope^18^,^19^,^21^ with our experimental setup offers another future possibility to observe many-body correlations more directly in a spatially resolved manner.

We anticipate promising future applications of applying ultrashort coherent laser pulses to ultracold Rydberg gases. Using alternative excitation schemes, one may investigate beyond mean-field effects in Ramsey experiments with more complex Hamiltonians such as Heisenberg-type Hamiltonians of interest for polar molecules^9^,^10^ and atomic clocks^57^ in optical lattices. Another application could be the investigation of a scenario in which Rydberg electronic wave functions are spatially overlapped between neighbouring Rydberg atoms^58^. This could lead to new exotic phases in which the Rydberg electrons are shared among many nuclei, and exchange interactions play key roles in their dynamical properties on the ultrafast timescales. Such a metal-like many-body Rydberg state would naturally lead to Penning ionization quite rapidly. However, Jaksch and colleagues^59^ have theoretically estimated the lifetime of such a metal-like Rydberg state of two ^85^Rb atoms (*v*=50) to be ∼100 ns. This is longer than our measurement timescale by more than two orders of magnitude, rendering the observation of this state possible using our time-domain approach.

## Methods

### Atom preparation

A magneto-optical trap (MOT) of ^87^Rb atoms was loaded from background vapour for 1.4 s. During the subsequent MOT compression for 30 ms, an optical dipole trap was turned on. The dipole trap was composed of a single 1,064 nm beam with its power and beam waist being ∼4 W and ∼30 μm (1/e^2^ radius), respectively. Polarization gradient cooling was performed for 100 ms. The trapped atoms were then transferred into the *F*=1 ground state by switching off the MOT repump laser. After that, we turned off the MOT trapping beams and the magnetic field. While keeping the intensity of the dipole trap laser, plain evaporative cooling was carried out for 50 ms. During the evaporation process, a 76 μT homogeneous magnetic field was turned on, pointing along the direction of the dipole trap laser. For the next 200 μs, the atoms were optically pumped to the 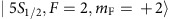 state by using the MOT repump beam and a 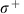 beam, which is resonant to the transition from 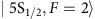 to 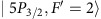 and counterpropagates with the dipole trap laser. The dipole-trap laser was turned off 2 μs before the irradiation of the picosecond infrared and blue pulses to avoid 2+1 multiphoton ionization induced by a combination of the picosecond pulses and the trapping laser beam, whereas the homogeneous magnetic field remained on. The picosecond infrared and blue pulses at ∼779 and ∼481 nm, which propagated collinearly with the dipole trap beam, had cross-sections with FWHMs of ∼130(100)μm and 30(30)μm along the *x* (*y*) direction (Fig. 1a).

### Estimation of the atom density

At first, the atom density in the Ramsey measurements was estimated solely from the total number of atoms and the size of the atomic ensemble obtained by *in-situ* absorption imaging with a CCD (charge-coupled device) camera without expanding the atomic ensemble. However, in contrast to the axial size of the atomic ensemble (∼2 mm FWHM), the spatial resolution of the *in-situ* absorption imaging with the CCD camera was not high enough mostly because of the aberrations of the imaging lens to resolve the radial size, resulting in an underestimation of the atom density.

In a later independent experiment, hereafter referred to as a ‘reference experiment', we estimated the radial size from the temperature of the atomic ensemble and the trap frequency of the radial direction, setting the trapping conditions almost the same as those employed in the Ramsey measurements. Those trapping conditions set almost the same were (1) the same loading sequence as described in the preceding subsection; (2) the dipole-trap laser power (with an error described in the next sentence); (3) the dipole-trap laser focusing; and (4) the total number of atoms (with an error described in the next sentence). We performed two reference experiments under two different trapping conditions corresponding to the higher and lower densities in the Ramsey measurements, setting the dipole-trap laser power (the trapping condition (2)) with a difference of ∼2% and ∼5% from the power averaged over the Ramsey measurements for the higher and lower densities, respectively, and setting the total number of atoms (the trapping condition (4)) within 1 s.d. ∼14% and ∼33% of the eight and seven corresponding values measured in the Ramsey measurements for the higher and lower densities, respectively. In these reference measurements, the temperature was measured by an expansion of the atomic ensemble and parametric heating was employed to infer the radial trap frequency. The temperatures and the trap frequencies were thus obtained to be ∼67 μK and 2.2 kHz and ∼39 μK and 1.1 kHz under those two trapping conditions, respectively. These temperatures and trap frequencies gave typical radial sizes (FWHM) of the atomic ensembles in the reference experiments to be ∼14 and 20 μm, which we regarded to be the radial sizes of the atomic ensembles in the Ramsey measurements for the higher and lower densities, respectively.

The total number of atoms was also slightly underestimated by the *in-situ* absorption imaging in the Ramsey measurements because of the spatial resolution, so that it was calibrated in a later independent experiment in which the loading sequence and the trap laser focusing (the trapping conditions (1) and (3)) were almost the same as those employed in the Ramsey measurements. In this calibration experiment, we measured the total number of atoms by the absorption imaging with the expansion of the atomic ensemble as a function of the one measured without the expansion to obtain a linear calibration curve with its slope being 1.04±0.03. This linear calibration curve gave the total numbers of atoms to be ∼6 × 10^5^ and ∼4 × 10^4^ for the higher and lower densities in the Ramsey measurements, respectively, for *v*=42.

These radial sizes and the total numbers of atoms were combined with the axial sizes measured *in situ* in the Ramsey measurements, to give the peak atom densities of ∼1.3 × 10^12^ and ∼4 × 10^10^ cm^−3^ for the higher and lower densities, respectively, for *v*=42. Similarly, the peak atom density and the total number of atoms for *v*=38 were estimated to be ∼1.2 × 10^12^ cm^−3^ and ∼5 × 10^5^ for the higher density and ∼4 × 10^10^ cm^−3^ and ∼4 × 10^4^ for the lower density, respectively, and for *v*=50 they were estimated to be ∼1.2 × 10^12^ cm^−3^ and ∼4 × 10^5^ for the higher density and ∼3 × 10^10^ cm^−3^ and ∼3 × 10^4^ for the lower density, respectively.

### Estimation of the temperature

The temperature of the atomic ensemble was not measured *in situ* in a series of the Ramsey measurements; however, it was measured in a later independent experiment, hereafter referred to as a ‘temperature experiment', in which we set the trapping conditions (1)(4) in the same way as described in the preceding subsection. In this temperature experiment, the temperature was measured by an expansion of the atomic ensemble. Two temperature experiments were performed independently under the trapping conditions corresponding to the higher density in the Ramsey measurements, giving ∼67 and ∼72 μK, respectively, so that we estimated the temperature of the higher density ensemble to be ∼70 μK. Similarly, a temperature experiment was performed independently under the trapping conditions corresponding to the lower density in the Ramsey measurements, giving ∼39 μK, so that we estimated the temperature of the lower density ensemble to be ∼39 μK.

### Rydberg excitation and detection

The output of a Ti:sapphire laser system (Spectra Physics; Spitfire Ace, wavelength ∼779 nm, pulse width ∼1 ps, repetition rate 1 kHz) was used as the infrared pulse and was also used to pump an optical parametric amplifier (Spectra Physics; TOPAS) to generate the blue pulse tuned to ∼481 nm. The repetition rate was reduced using pulse pickers to synchronize it with the atom preparation sequence whose duration was 1.6 s. To reduce the number of Rydberg states to be excited, the spectra of infrared and blue pulses were cut using homemade pulse shapers in a 4*f*-setup (*f*=500 mm), respectively. The infrared and blue pulses were combined collinearly with a dichroic mirror and their relative timing was coarsely adjusted to be zero by cross-correlation measurements based on sum frequency generation and was further optimized to maximize the Rydberg ion signals. They were introduced into a Michelson-type interferometer to produce a pair of identical double pulses, each of which was composed of the infrared and blue pulses. Those two double pulses induced the pump and probe excitations, respectively (see Fig. 1a). The relative phase between these two double pulses were tuned with attosecond precision^48^. Those two double pulses were combined collinearly with the dipole-trap laser beam with another dichroic mirror. Those two double pulses and the trapping laser beam were focused with a plano-convex lens (*f*=250 mm) to the atomic ensemble.

The infrared and blue pulses, and the optical pumping beam were circularly polarized in the same direction with respect to the magnetic field, so that the state 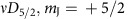 was mostly populated, and excitations to *S* Rydberg states were suppressed due to transition selection rules even though the effective two-photon excitation spectrum covered the *S* states. This excitation scheme also suppressed the Raman transition between the *F*=2 and *F*=1 hyperfine states in the ground state that could happen within the single infrared pulse to induce undesirable beating in the Ramsey contrast as a function of *τ*, of which an example is shown in Fig. 2d, with a period of 146 ps, which is the reciprocal of the hyperfine splitting 6.83 GHz.

The maximum population of the *vD* Rydberg states was not >5%, to suppress photo-ionization. The typical pulse energies of the infrared and blue pulses were ∼10 and 400 nJ, respectively, for the 1.2% population and were ∼30 and 600 nJ, respectively, for the 3.3% population. The population was estimated from the loss of the number of the ground-state atoms in the higher-density ensemble induced by the irradiation of the picosecond infrared and blue pulses. We measured the number of the atoms by absorption imaging with and without the picosecond pulses and compared those numbers to evaluate the loss. See the previous subsection ‘Estimation of the atom density' for the details of the evaluation of the atom number by absorption imaging. The loss induced by a single pair of the infrared and blue pulses was too small (a few percent) to be evaluated securely, so that we shined 30 or 50 pairs of the infrared and blue pulses, each of which was accompanied by field ionization^16^, at a 1 kHz repetition rate to induce the loss that was clearly visible and was used to infer the loss induced by the single pair of the infrared and blue pulses. The loss for different amounts of pulse pairs followed the relation 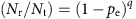, where *N*_r_ is the number of atoms remaining after *q* pulses and *N*_t_ is the total atom number. *p*_e_ is the Rydberg state population for the atoms in the ensemble.

After the probe excitation, the populated Rydberg states were ionized by means of field ionization. The Rb^+^ ions thus produced were detected with a micro channel plate placed 5.5 cm away from the atomic ensemble. The electric field for the ionization was triggered 50 ns after the probe excitation, reaching the ionization threshold within the next 100 ns. The output of the micro channel plate was amplified with a preamplifier and sent to a gated integrator, whose output was fed into a computer. It is noteworthy that the field-ionization spectrum shown in Fig. 1c was measured using an oscilloscope with the electric field ramped up slowly on the microsecond timescale to check how many Rydberg states were populated by our broadband excitation with the picosecond pulses.

### Time-domain Ramsey interferometry

The interaction among Rydberg atoms was observed by time-domain Ramsey interferometry with a pair of two-photon excitations: pump and probe. Their delay *τ* was coarsely tuned on the picosecond timescale with a motorized mechanical stage placed in one arm of the interferometer mentioned above and was scanned finely on the attosecond timescale with a piezoelectric transducer to measure Ramsey interferograms. A He-Ne laser beam was introduced to the interferometer to check the linearity of the scan by monitoring its optical interference. The period of this optical interference was also used for the calibration of the pump-probe delay *τ*. We measured the field-ionization signals of the two atomic ensembles with different densities alternately to suppress systematic uncertainties, scanning *τ* in steps of ∼30 as over a range of ∼3 fs at each coarse delay tuned by the mechanical stage on the picosecond timescale. The obtained Ramsey interferogram was fitted with a sinusoidal function. As the expected energy shift induced by the interaction was at most on the order of 10 GHz, much smaller than the eigenfrequency of the Rydberg state itself (∼1 × 10^15^ Hz), we fitted the interferograms with the same eigenfrequencies for the higher and lower densities to evaluate the phase shift of the higher-density ensemble from the lower-density one. We have defined the contrast of the interferogram to be the ratio of the amplitude of the fitted sinusoidal function to its mean value. In Fig. 2a–c, the signal intensities are normalized by the mean value of the sinusoidal function fitted to each interferogram. We have defined Ramsey contrast to be the ratio of the contrast of the higher-density ensemble to that of the lower-density one, as shown in Figs 3, 4 and 6. We assume that the interactions are negligible in the lower-density ensemble, which is thus taken to be a reference for measuring the contrast decay and the phase shift that may be induced by the interactions in the higher-density ensemble.

### Outline of the exactly solvable model simulation

We represent each atom as a two-level system, which is a pseudo-spin system, consisting of a ground state |g〉 (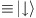) and an excited Rydberg state |e〉 (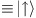) with energies *E*_g_ and *E*_e_, respectively. This assumption should be reasonable, as the contribution of the neighbouring Rydberg levels is small for the 42*D*_5/2_ state, as seen from the field-ionization spectrum shown in Fig. 1c. The *N*-atom wave function 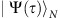 is initially assumed to be a product of independent single-atom wave functions 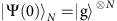. The experiment consists of four stages: (i) the pump excitation, (ii) an evolution with an *N*-atom Hamiltonian *H*, (iii) the probe excitation and (iv) the population measurement by field ionization. The experimental observable is the number of Rydberg excited atoms detected as a time-dependent signal 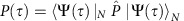, which is the expectation value of the sum of projection operators 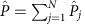, where 
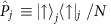
 measures the population in the Rydberg state for atom *j* normalized to the total atom number *N*.

The *N*-atom Hamiltonian in stage (ii) includes the atomic energies and the interactions 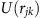 between atoms in the Rydberg states. This is justified on the picosecond timescale of the current measurement, as Rydberg interactions provide coupling strengths on the order of GHz for micrometre separations, much larger than those between Rydberg- and ground-state atoms and between two ground-state atoms on the order of kilohertz and less than kilohertz, respectively, for micrometre separations. This leads to a diagonal Hamiltonian of the form in equation (2).

### Continuum approximation

In this approximation we assume the density distribution to be homogeneous in a small volume around a particular position **r** (ref. 54). The Ramsey signal *P*(**r**, *τ*) is calculated for such a homogeneous region as


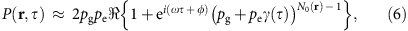


where 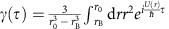 and *r*_0_ is referred to as a cutoff radius, so that we consider interactions only among the atoms within a sphere whose radius is *r*_0_. A blockade radius *r*_B_ is determined by the bandwidth of the pump excitation and is ∼0.88 μm in the present experiment. *N*_0_(**r**) is the number of atoms in the local volume 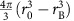 and depends on the local density *n*(**r**) due to 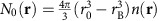. This signal is averaged over the whole ensemble whose atom-density distribution is assumed to be Gaussian with the peak atom-density *n*_p_ to obtain equation (4), in which the Ramsey contrast and phase shift can be derived from


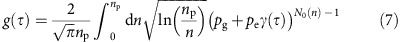


to be the absolute value and phase of the function *g*(*τ*), respectively. In the case that the potential is given by a van der Waals interaction and in the limit 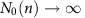, the continuum approximation leads to the expression in equation (5) for the function *g*(*τ*). We expect this approximation to describe the system well for a sufficiently low population of Rydberg excited atoms *p*_*e*_<<*p*_*g*_ (ref. 54), which is safely satisfied in our experiments.

### Data availability

The data that support the findings of this study are available from the corresponding author upon request.

## Additional information

**How to cite this article:** Takei, N. *et al*. Direct observation of ultrafast many-body electron dynamics in an ultracold Rydberg gas. *Nat. Commun.* **7,** 13449 doi: 10.1038/ncomms13449 (2016).

**Publisher's note:** Springer Nature remains neutral with regard to jurisdictional claims in published maps and institutional affiliations.

## Supplementary Material

Supplementary Information Supplementary Figures 1-13, Supplementary Notes 1-10 and Supplementary References.
