# Supplementary material for: Direct observation of ultrafast many-body electron dynamics in an ultracold Rydberg gas
Source: Nat Commun. 2016 Nov 16;7:13449. doi: 10.1038/ncomms13449 (PMC5116092; doi:10.1038/ncomms13449)
Supplement: Supplementary Information — Supplementary Figures 1-13, Supplementary Notes 1-10 and Supplementary References. [file ncomms13449-s1.pdf]

Supplementary Figures

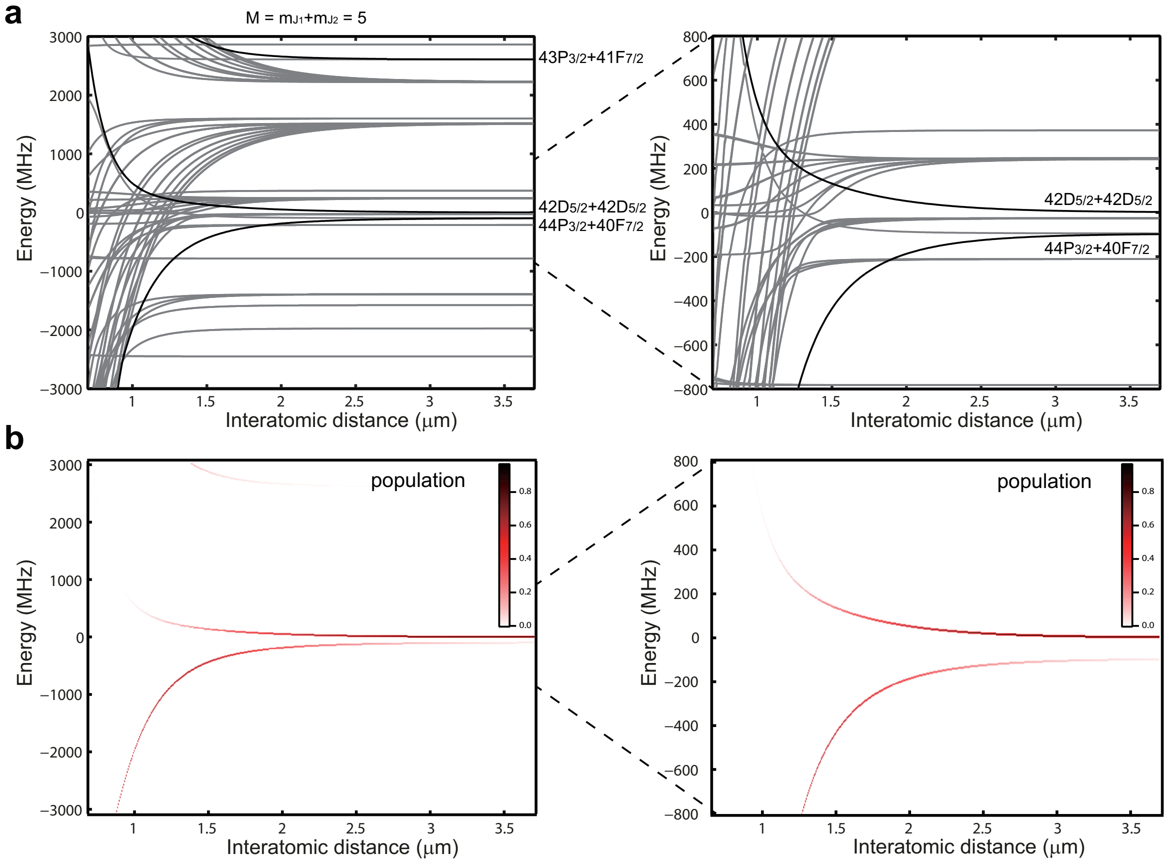

Supplementary Figure 1: **Two-atom dipolar potentials around the $\mathbf{42D_{5/2} + 42D_{5/2}}$ asymptote.** $\theta = 0$ and $M = m_{J_1} + m_{J_2} = 5$, where θ is the same angle as has been introduced in Supplementary Eq. (20). Only the asymptotic states with electronic angular-momenta below $l = 5$, principal quantum-numbers ranging from 38 to 46, and within ±30 GHz from the $42D_{5/2} + 42D_{5/2}$ asymptote are considered in the diagonalization of the potential-energy matrix. (**a**) The potentials that dominate the interaction between two $|42D_{5/2}, m_J = 5/2\rangle$ atoms are indicated by black solid lines. (**b**) The color code indicates the fraction of the population of the asymptotic $|42D_{5/2}, m_J = 5/2\rangle \otimes |42D_{5/2}, m_J = 5/2\rangle$ state that is contained in an interaction-induced mixed state as a function of the interatomic distance. Only the contribution of the $|42D_{5/2}, m_J = 5/2\rangle \otimes |42D_{5/2}, m_J = 5/2\rangle$ asymptotic state needs to be considered since it is the only state in the displayed energy range that can be addressed by the pulse excitation due to selection rules.

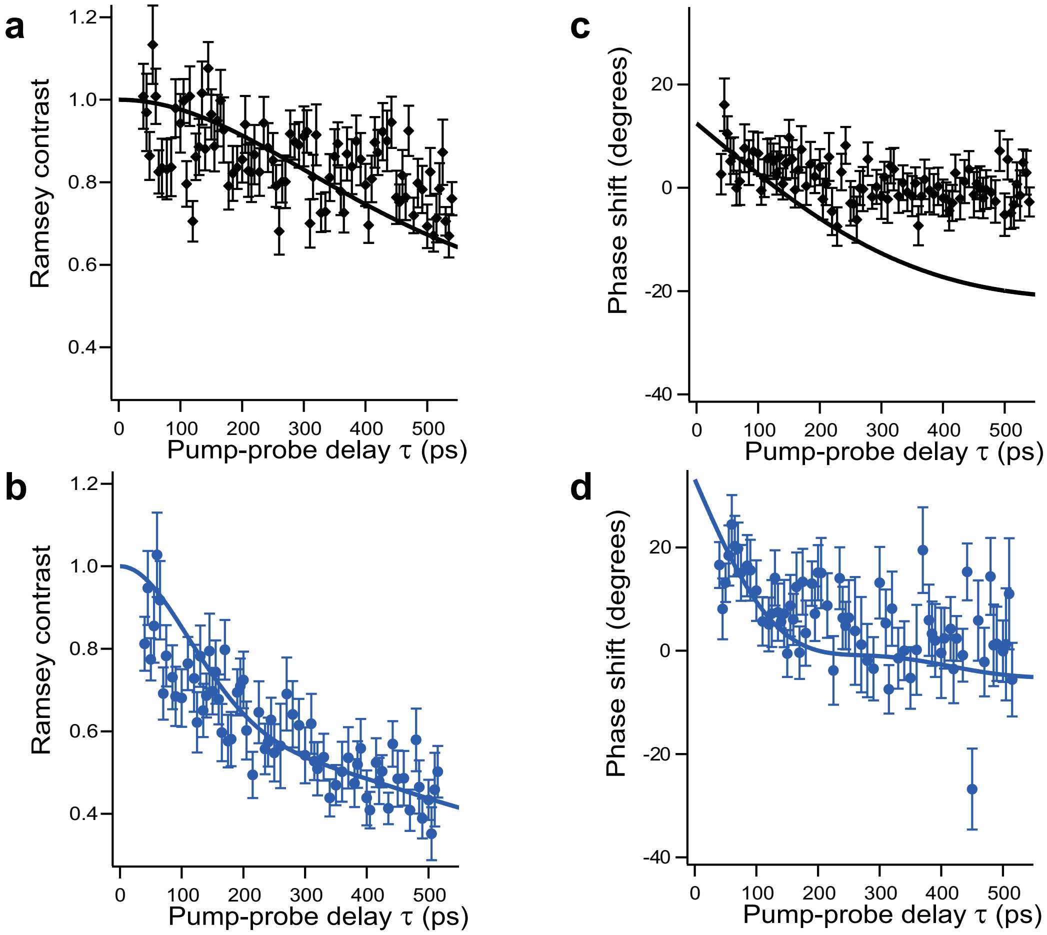

Supplementary Figure 2: **Mean-field analysis of the Ramsey contrast and phase-shift with an anisotropic van der Waals potential.** The black-diamond-shaped and blue-circle data-points show the Ramsey contrasts (**a** and **b**) and the phase-shifts (**c** and **d**) measured with the population of the $42D_{5/2}$ state being ~1.2% (**a** and **c**) and ~3.3% (**b** and **d**), respectively. In **a** and **b**, the Ramsey contrasts simulated by the mean-field model with the anisotropic potential given by Supplementary Eq. (20) (black and blue solid lines) are compared with the measured ones. Similarly the measured and simulated phase-shifts are compared in **c** and **d**. The interaction strength is limited below 75 GHz, which is the bandwidth (half width half maximum) of the pump excitation, and the peak atom density is set to $\sim 1.3 \times 10^{12}$ cm^{-3} in these simulations. The coefficient $C_6 = 3.4\,\mathrm{GHz}\,\mu\mathrm{m}^6$ has been used in these simulations. The error bars represent the standard deviation.

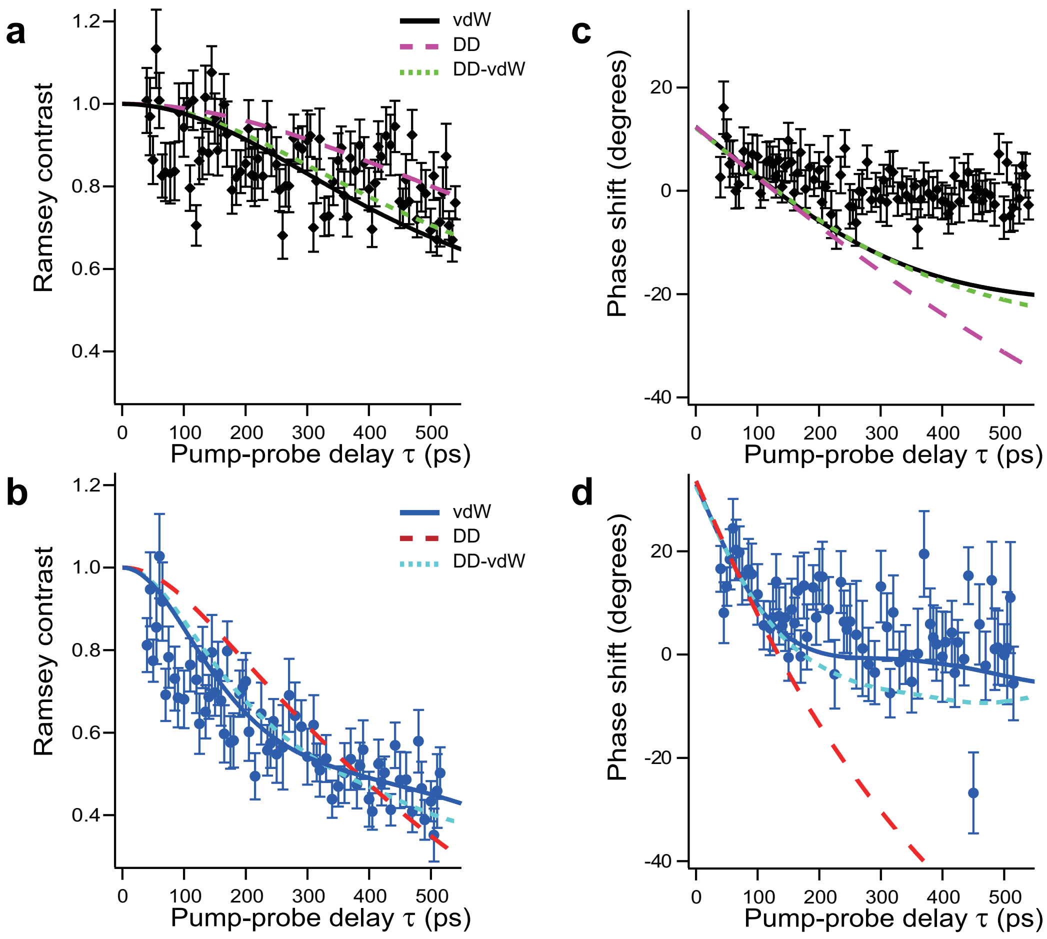

Supplementary Figure 3: **The mean-field analysis of the Ramsey contrast and phase-shift with the dipole-dipole (DD) interaction and the hybrid form of a dipole-dipole and a van der Waals (DD-vdW) interaction without anisotropies.** The black-diamond-shaped and blue-circle data-points show the Ramsey contrasts (**a** and **b**) and the phase-shifts (**c** and **d**) measured with the population of the $42D_{5/2}$ state being ~1.2% (**a** and **c**) and ~3.3% (**b** and **d**), respectively. In **a** and **b**, the Ramsey contrasts are simulated by the mean-field model with the DD (dahed line) and DD-vdW (dotted line) interactions without anisotropies and are compared with the measured ones. Similarly the measured and simulated phase-shifts are compared in **c** and **d**. The mean-field simulations with the pure van der Waals (vdW) interaction (solid line), which have been shown in Fig. 3 in the main text, are presented again to be compared with the DD and DD-vdW result. The interaction strength is limited below 75 GHz, which is the bandwidth (half width half maximum) of the pump excitation, and the peak atom density is set to $\sim 1.3 \times 10^{12}\,\mathrm{cm}^{-3}$ in these simulations. The coefficient $C_3 = 1\,\mathrm{GHz}\,\mu\mathrm{m}^3$ has been used for the DD interaction, and a combination of $C_3 = 3.4\,\mathrm{GHz}\,\mu\mathrm{m}^3$ and $r_c = 0.81\,\mu\mathrm{m}$ have been used for the DD-vdW interaction [see Supplementary Eq. (21)]. The error bars represent the standard deviation.

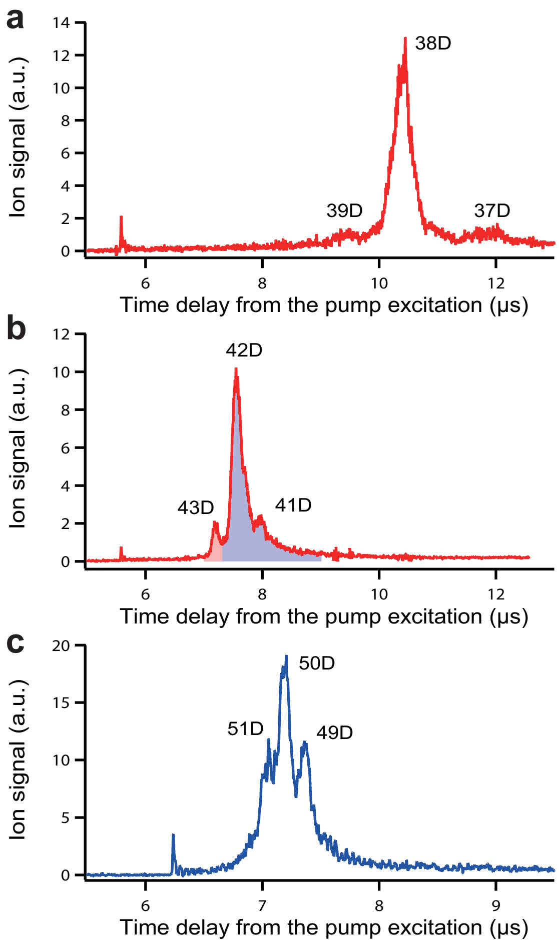

Supplementary Figure 4: **State-resolved field-ionization spectra.** (**a**) Ion signal measured as a function of time-delay from the ps pulsed-laser excitations to Rydberg states $\nu = 38$. The electric field was ramped at the same speed as the one for Fig. 1c in the main text. The atom density and Rydberg population were $\sim 4 \times 10^{10}\,\mathrm{cm}^{-3}$ and $3.2 \pm 0.1\,\%$, respectively. (**b**) The ion signal for $\nu = 42$ with the atom density and Rydberg population being $\sim 4 \times 10^{10}\,\mathrm{cm}^{-3}$ and $1.2 \pm 0.1\,\%$, respectively. This is the same signal as shown in Fig. 1c. The red-shaded region indicates the integration range used for the estimation of the relative population in the 43D state, whereas the blue-shaded area shows the integration range for the 42D and 41D states. (**c**) The ion signal for $\nu = 50$ with the atom density and Rydberg population being $\sim 3 \times 10^{10}\,\mathrm{cm}^{-3}$ and $3.1 \pm 0.2\,\%$, respectively. The ramp-up speed of the electric field was slower than that for $\nu = 42$ and 38 by a factor of 2/3. The bandwidths of the excitations in these measurements in **a** – **c** are the same as the one for the pump excitation in the Ramsey measurements.

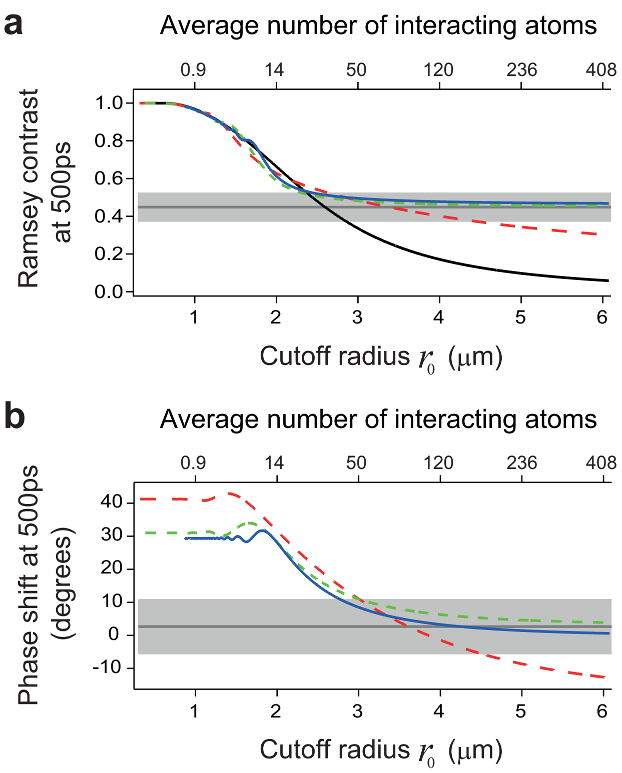

Supplementary Figure 5: **Convergence of the simulated Ramsey contrast and phase-shift as functions of an average number of interacting atoms.** The Ramsey contrast (**a**) and phase-shift (**b**) at τ = 500 ps are simulated by the theory model with the continuum approximation and are plotted as functions of the cutoff radius r_0 (the lower abscissa) and of an average number of interacting atoms within the volume $V = \frac{4\pi}{3}(r_0^3 - r_B^3)$ (the higher abscissa). The population of the $42D_{5/2}$ is set to $\sim 3.3\,\%$ in these simulations. The interaction strength is limited below 75 GHz, which is the half width half maximum of the pump excitation, and the peak atom density is set to $\sim 1.3 \times 10^{12}\,\mathrm{cm}^{-3}$ in these simulations. The red dashed and green dotted lines show the results with the dipole-dipole interaction and the hybrid form of a dipole-dipole and a van der Waals interaction without anisotropies, respectively. The results with the van der Waals interaction without an anisotropy, which has been used in the main text, are displayed by the blue solid lines. The dark-grey solid lines represent the measured Ramsey contrast and the phase-shift, each of which is the average over eight points around τ = 500 ps in Figs. 6b or d. The light-grey shaded area represents one standard deviation of the average over those eight measured values. The black solid line shows the Ramsey contrast $|g(\tau)|$ given by Eq. (7) in the main text with $\gamma(\tau) = 0$, giving the upper limit of the contrast decay and accordingly the lower limit of the number of atoms ~ 32 to reproduce the Ramsey contrast ~ 0.45 measured at τ = 500 ps, irrespective of the potential curves.

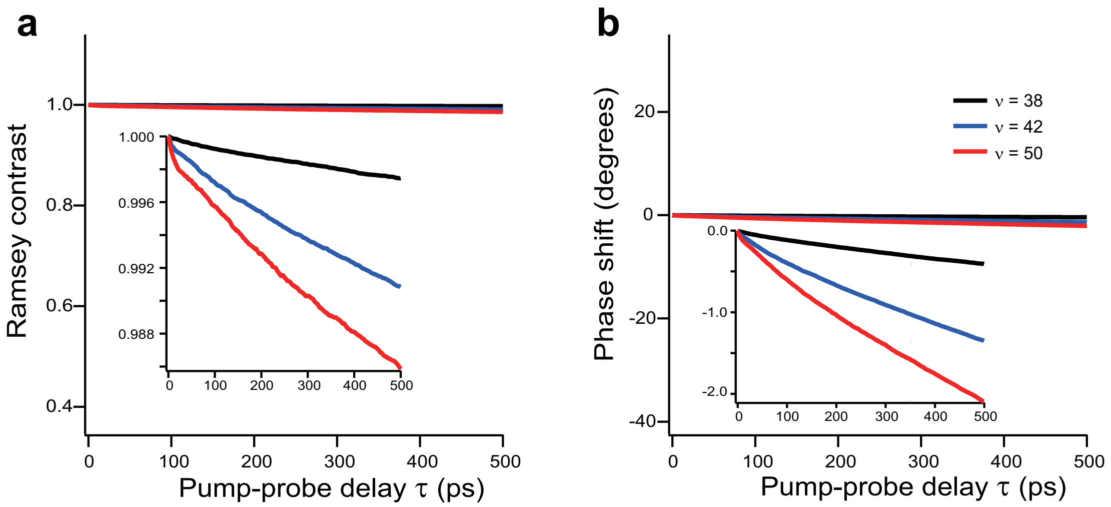

Supplementary Figure 6: **Numerical simulation of the photo-ions contribution to the Ramsey signal.** (**a**) Ramsey-contrasts simulated as functions of pump probe delay τ in the presence of photo-ions for ν = 38 (black), 42 (blue), and 50 (red). (**b**) Phase-shifts simulated as functions of pump probe delay τ in the presence of photo-ions for ν = 38 (black), 42 (blue), and 50 (red).

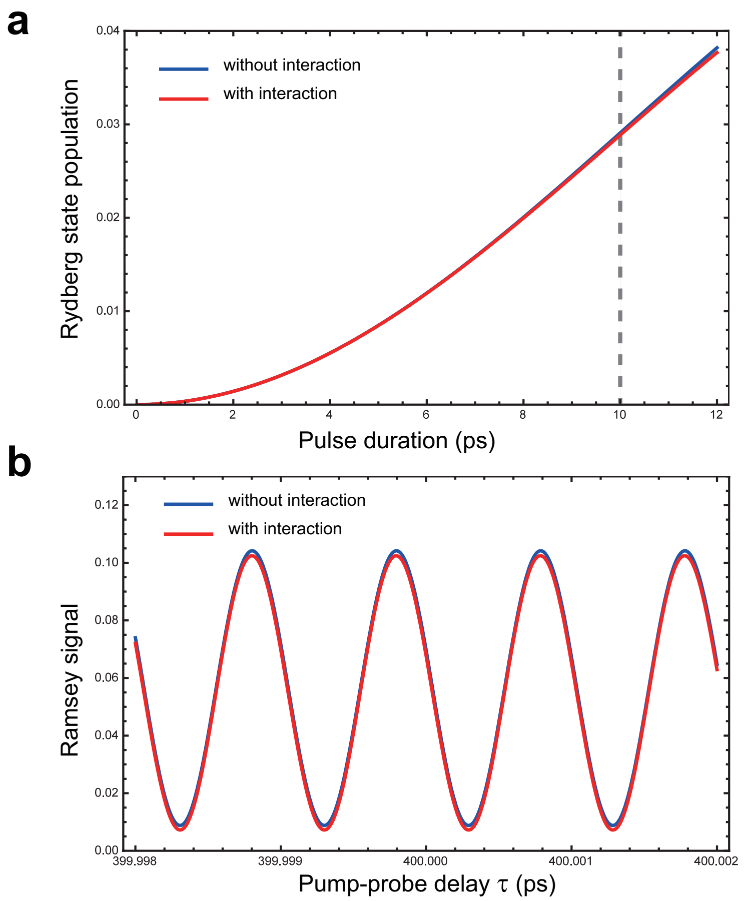

Supplementary Figure 7: **Simulation of the effects of the Rydberg interactions during the excitation on the Ramsey signal.** (**a**) The Rydberg populations at the end of the excitation pulse as functions of the pulse duration with (red) and without (blue) the interaction. (**b**) the Ramsey oscillations around the pump-probe delay $\tau \sim 400\,\mathrm{ps}$ with (red) and without (blue) the interaction during the excitation pulses.

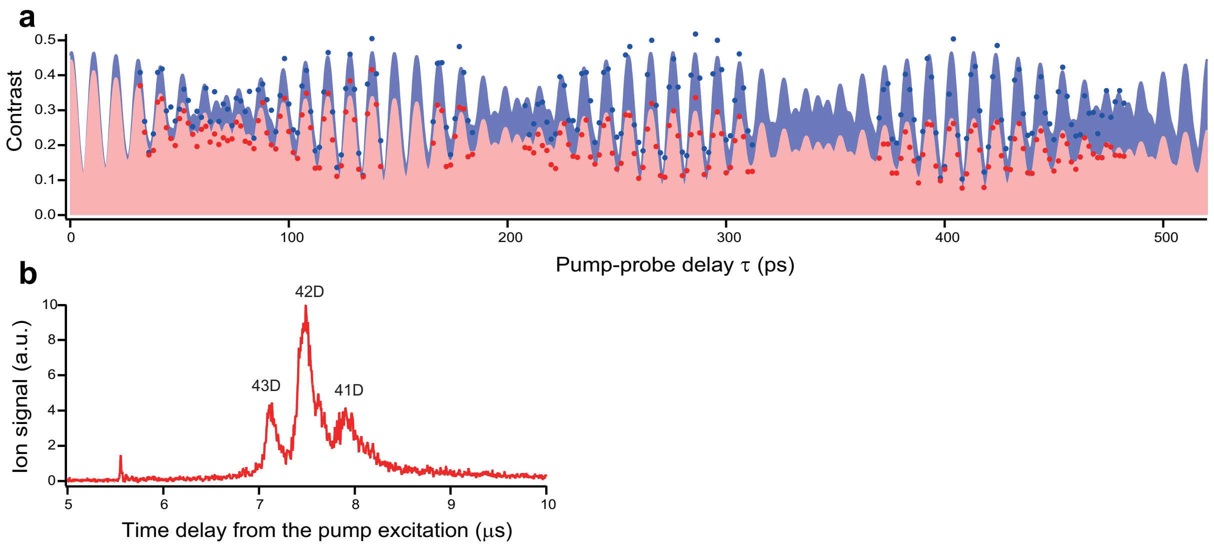

Supplementary Figure 8: **High resolution measurement of the Ramsey-oscillation contrasts.** (**a**) The contrasts as functions of the pump-probe delay τ for the higher-density (red dots) and lower-density (blue dots) ensembles, respectively. The red- and blue-shaded parts are numerically simulated recurrence-motions of the Rydberg wave-packet for the higher- and lower-density ensembles, respectively. (**b**) Field ionization spectrum associated with the measurement shown in **a**.

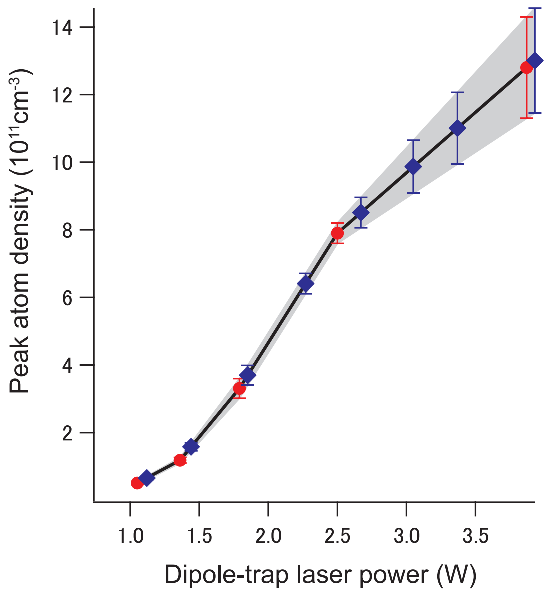

Supplementary Figure 9: **The calibration of the peak atom density as a function of the power of the dipole-trap-laser.** The red-circle data points show the peak atom density measured in the calibration experiment as a function of the dipole-trap-laser power. The blue-diamond-shaped data and their error bars are not measured directly, but are obtained by linear interpolation. These blue-diamond-shaped data points represent the estimated peak atom densities used in the density dependence measurement of the Ramsey contrast shown in Fig. 4b in the main text. The error bars represent one standard deviation.

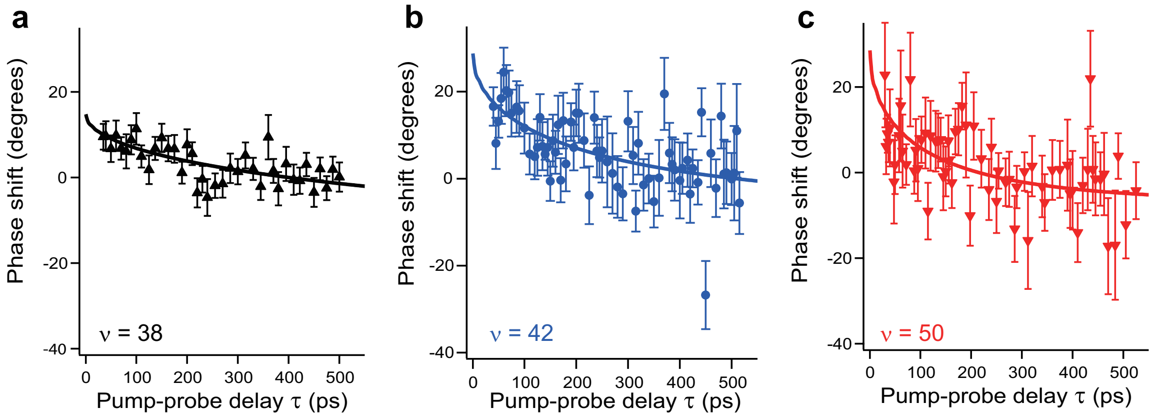

Supplementary Figure 10: **The principal-quantum-number dependences of the phase-shift.** (**a** – **c**) Measured phase-shifts are plotted as functions of τ for three different Rydberg levels ν = 38, 42, and 50. Each of them has been measured simultaneously with the Ramsey contrast with the same principal-quantum-number shown in Fig. 4a in the main text. The simulations indicated by the black, blue, and red solid-lines have been performed by the theory model with the continuum approximation for the van der Waals interaction with the adjusting parameters being $C_6 = 8\,\text{GHz}\,\mu\text{m}^6$ for $\nu = 38$, $C_6 = 34\,\text{GHz}\,\mu\text{m}^6$ for $\nu = 42$, and $C_6 = 103\,\text{GHz}\,\mu\text{m}^6$ for $\nu = 50$. The peak atom-density is set to the estimated density for each Rydberg level in these simulations. The density estimations are described in Methods section "Estimation of the atom density" in the main text. It should be noted that several Rydberg states are excited in the case of $\nu = 50$, whereas we have considered an excitation only to a single Rydberg state to perform the simulations for all of the three Rydberg levels. The error bars represent the standard deviation.

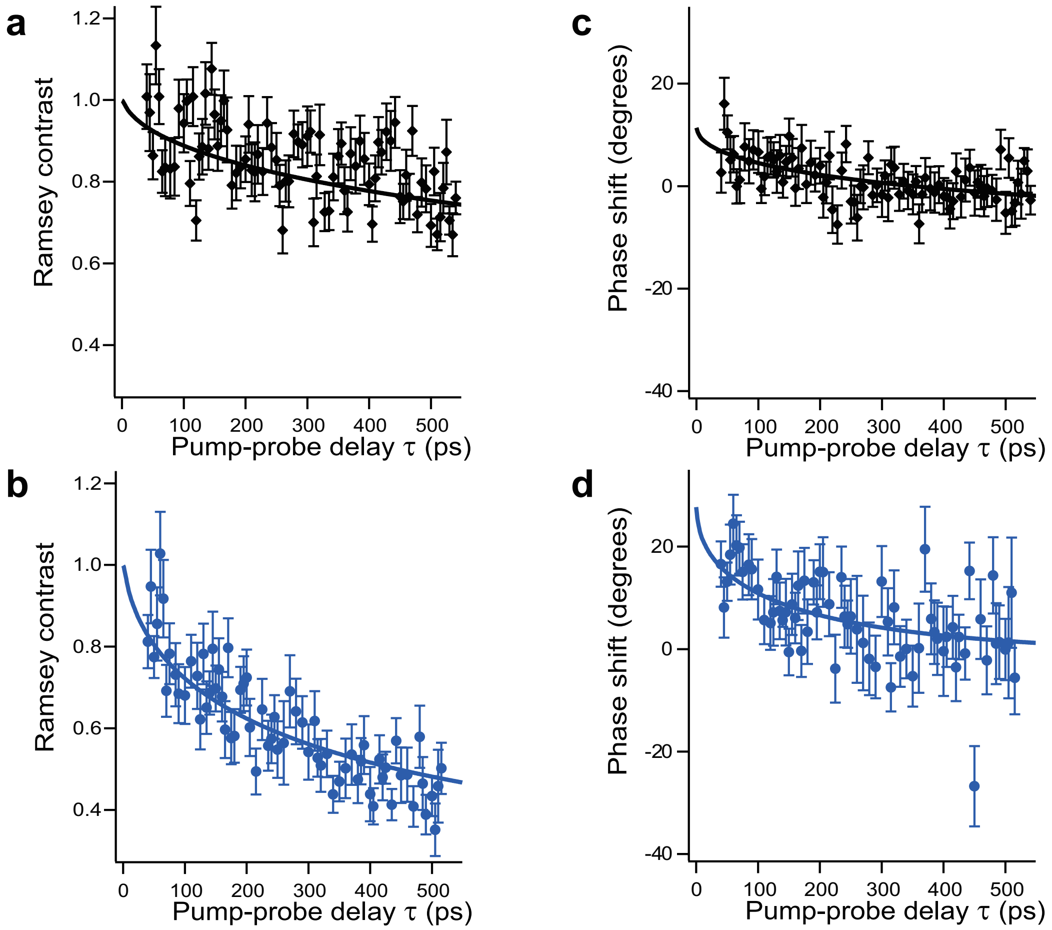

Supplementary Figure 11: **The theory-model analysis of the Ramsey contrast and phase-shift beyond mean-field with the continuum approximations and with an anisotropic van der Waals potential.** The black-diamond-shaped and blue-circle data-points show the Ramsey contrasts (**a** and **b**) and the phase-shifts (**c** and **d**) measured with the population of the $42D_{5/2}$ state being ∼1.2% (**a** and **c**) and ∼3.3% (**b** and **d**), respectively. In **a** and **b**, the Ramsey contrasts simulated with the anisotropic potential given by Supplementary Eq. (20) (black and blue solid lines) are compared with the measured ones. Similarly, the measured and simulated phase-shifts are compared in **c** and **d**. These simulated results have been obtained by Eq. (6) in the main text combined with the potential anisotropy, employing the cutoff radius $r_0 \sim 4\,\mu$m and its corresponding atom-number $N_0 = 450$ at the peak density. The interaction strength in these simulations is limited below 75 GHz, which is the half width half maximum of the pump excitation, and the peak atom density is set to $\sim 1.3 \times 10^{12}\,\mathrm{cm}^{-3}$ in these simulations. The coefficient $C_6 = 63\,\mathrm{GHz}\,\mu\mathrm{m}^6$ has been used in these simulations. The error bars represent the standard deviation.

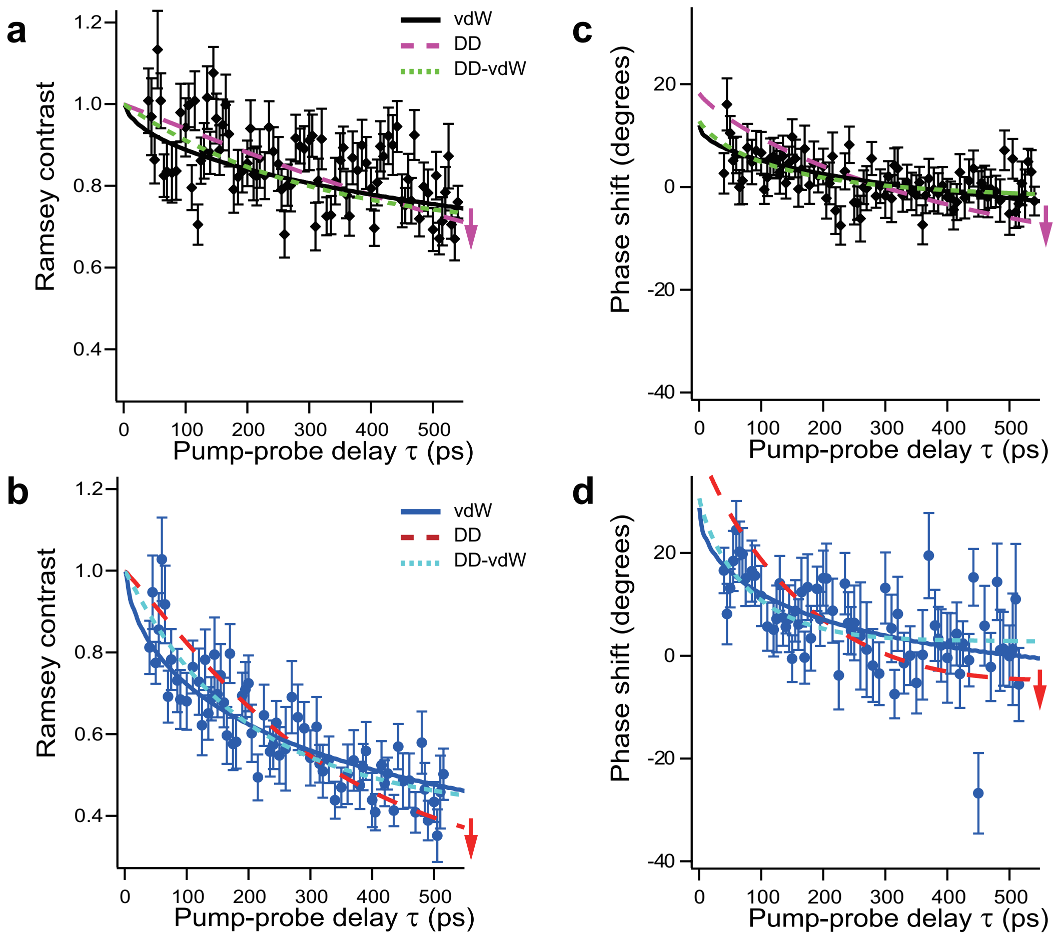

Supplementary Figure 12: **The theory-model analysis of the Ramsey contrast and phase-shift beyond mean-field with the continuum approximation and with the dipole-dipole (DD) interaction and the hybrid form of a dipole-dipole and a van der Waals (DD-vdW) interaction without anisotropies.** The black-diamond-shaped and blue-circle data-points show the Ramsey contrasts (**a** and **b**) and the phase-shifts (**c** and **d**) measured with the population of the $42D_{5/2}$ state being ∼1.2% (**a** and **c**) and ∼3.3% (**b** and **d**), respectively. In **a** and **b**, the Ramsey contrasts simulated with the DD (dahed line) and DD-vdW (dotted line) interactions without anisotropies are compared with the measured ones. Similarly the measured and simulated phase-shifts are compared in **c** and **d**. The simulated results for the DD interaction have been obtained by Eq. (6) in the main text, employing the cutoff radius $r_0 \sim 4\,\mu$m and its corresponding atom-number $N_0 = 450$ at the peak density. It should be noted that these DD results are pushed down as the cutoff radius is further increased, as is indicated by arrows in the figures and will be further discussed with Supplementary Fig. 5. The simulations with the pure van der Waals (vdW) interaction (solid line), which have been shown in Fig. 6 in the main text, are presented again to be compared with the DD and DD-vdW results. The interaction strength is limited below 75 GHz, which is the bandwidth (half width half maximum) of the pump excitation, and the peak atom density is set to $\sim 1.3 \times 10^{12}\,\mathrm{cm}^{-3}$ in these simulations. The coefficient $C_3 = 2.5\,\mathrm{GHz}\,\mu\mathrm{m}^3$ have been used for the DD interaction, and a combination of $C_3 = 4.3\,\mathrm{GHz}\,\mu\mathrm{m}^3$ and $r_c = 1.93\,\mu$m have been used for the DD-vdW interaction [see Supplementary Eq. (21)]. The error bars represent the standard deviation.

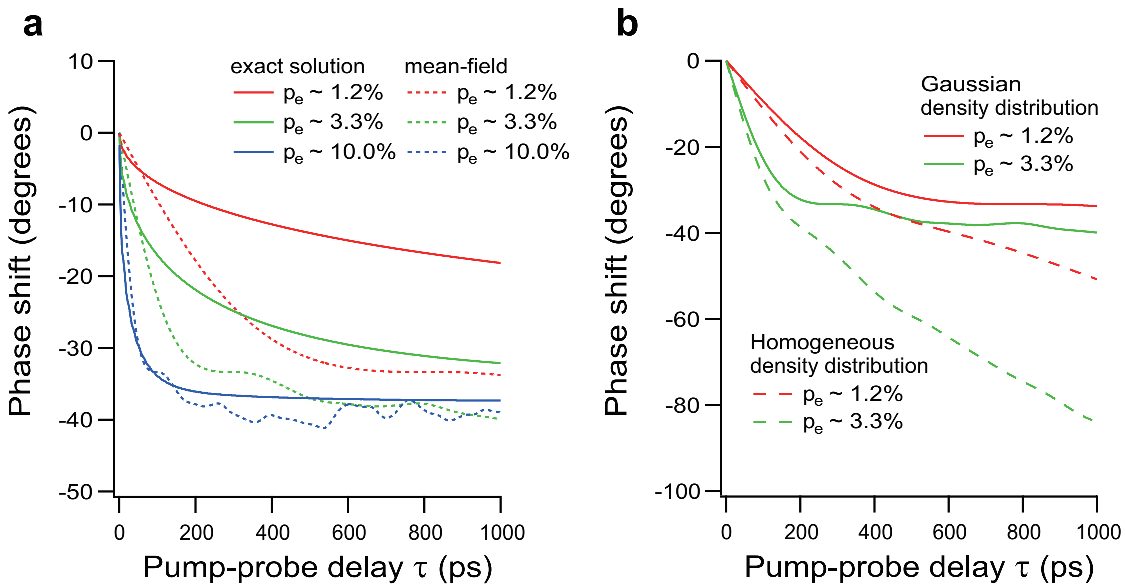

Supplementary Figure 13: **Phase-shifts calculated as functions of the pump-probe delay** τ**.** (**a**) Comparison between the exact (solid lines) and mean-field (dotted lines) solutions with the Gaussian atom-density distribution for different populations p_e ($\sim$ 1.2, $\sim$ 3.3, and $\sim$ 10.0 %) in the 42D Rydberg state. The Gaussian distribution has been taken from the Ramsey experiment shown in the main text. The C_6 coefficients are set to 34 GHz μm^6 for the exact solution and 1.9 GHz μm^6 for the mean-field solution, respectively, which are the values used in the calculations performed in the main text. (**b**) Comparison between phase-shifts calculated with the Gaussian (solid lines) and homogenous (dashed lines) atom-density distributions within the mean-field model for different populations $p_e \sim 1.2$ % and ~ 3.3 % in the 42D Rydberg state. The Gaussian distribution is the same as in **a**, whereas the homogeneous density has been set to the average density of the Gaussian distribution. The C_6 value is set the same as in **a**.

Supplementary Notes

Supplementary Note 1: Estimation of the photo-ion contribution to the Ramsey signal

In order to investigate the effect of the electric field generated by photo-ions on the Ramsey signal, we have performed numerical simulations for $\nu = 38$, 42, and 50, including ion contributions. In each of those simulations, atom locations in an ensemble with a Gaussian distribution are generated by the Monte-Carlo method. In this list of atom locations a certain number of random locations are taken to be ion locations. The number of ions is obtained as follows. The ion fractions are estimated for $\nu = 38$ and 50 from the field ionization spectra shown in Supplementary Figs. 4a and c, respectively, and for $\nu = 42$ from the spectrum similar to the one shown in Supplementary Fig. 4b to be the ratios of the areas of the ion peaks at $\sim 5.6\,\mu$s for $\nu = 38$, 42 and $\sim 6.2\,\mu$s for $\nu = 50$ to the total areas of the spectra. The numbers of ions are thus estimated as the products of those ion fractions, our Rydberg populations $p_{\rm e}$'s, and the total number of atoms to be 109 for $\nu = 38$ with $p_{\rm e} \sim 3.2\,\%$, 232 for $\nu = 42$ with $p_{\rm e} \sim 3.3\,\%$, and 108 for $\nu = 50$ with $p_{\rm e} \sim 3.1\,\%$, respectively. These numbers are the upper limits of the number of photo-ions as they may include the ions produced at much later time (100 ns timescale) by Rydberg interactions [1]. At each atom position in each of the ensembles with these ion numbers, the joint electric field generated by those ions is calculated. The Stark shift induced by this joint electric field is calculated for each atom, so that the period of its Ramsey oscillation is shifted accordingly. Those Ramsey oscillations are averaged over the whole ensemble of the Rydberg atoms to give the contrast-decay and phase-shift shown in Supplementary Fig. 6 for each of $\nu = 38$, 42, and 50. It is seen that the contrast-decay and phase-shift are less than 2 % and 2.5 degrees at the longest pump-probe delay 500 ps in our measurements and are almost negligible.

Supplementary Note 2: Discussion on the pump-probe excitation and the zero-delay offset in the phase-shift

We consider the time-domain Ramsey interferometry with the identical pump and probe excitations, each of which is a one-photon excitation of a two-level atom, consisting of a ground state $|\mathrm{g}\rangle$ and an excited state $|\mathrm{e}\rangle$, with rectangular laser pulses for simplicity. The results can be easily adapted to near-resonant two-photon excitations that have been used in the present experiments.

The time dependent Schrödinger equation is given by

$$\begin{aligned} i\hbar\frac{\partial}{\partial t}|\psi\rangle &= H|\psi\rangle \qquad (1)\\ &= \left(\hbar\frac{\omega}{2}|\mathrm{e}\rangle\langle\mathrm{e}| - \hbar\frac{\omega}{2}|\mathrm{g}\rangle\langle\mathrm{g}| - \mathbf{d}_{\mathrm{eg}}\cdot\mathbf{E}(t)|\mathrm{e}\rangle\langle\mathrm{g}| - \mathbf{d}^{*}_{\mathrm{eg}}\cdot\mathbf{E}(t)|\mathrm{g}\rangle\langle\mathrm{e}|\right)|\psi\rangle. \end{aligned}$$

Here, ω is the angular frequency for the atomic transition, $\mathbf{E}(t) = \mathbf{E}_0(\Theta(t-t_0) - \Theta(t-t_1))\cos(\omega_l(t-t_0))$ is the pump laser field where ω_{l} is the laser frequency, $\Theta(t)$ is the Heaviside step function, t_0 is the beginning of the pulse excitation, t_1 is the end of the excitation, $|\psi\rangle = C_{\mathrm{g}}(t)|\mathrm{g}\rangle + C_{\mathrm{e}}(t)|\mathrm{e}\rangle$ is the atomic state, and $\mathbf{d}_{\mathrm{eg}} = \langle\mathrm{e}|\mathbf{d}|\mathrm{g}\rangle$ is the dipole-matrix element. Solving the Schrödinger equation Eq. (1) by employing the rotating-wave approximation for the evolution of the atomic state during the pump excitation we obtain

$$\begin{aligned} \begin{pmatrix} C_{\mathrm{g}}(t_1) \\ C_{\mathrm{e}}(t_1) \end{pmatrix} &= \begin{pmatrix} (\cos(\theta/2) - i\eta\sin(\theta/2))e^{i\frac{\omega_{\mathrm{l}}}{2}\delta t} & -i\xi\sin(\theta/2))e^{i\chi}e^{i\frac{\omega_{\mathrm{l}}}{2}\delta t} \\ -i\xi\sin(\theta/2)e^{-i\chi}e^{-i\frac{\omega_{\mathrm{l}}}{2}\delta t} & (\cos(\theta/2) + i\eta\sin(\theta/2))e^{-i\frac{\omega_{\mathrm{l}}}{2}\delta t} \end{pmatrix} \begin{pmatrix} C_{\mathrm{g}}(t_0) \\ C_{\mathrm{e}}(t_0) \end{pmatrix} \\ &= A\begin{pmatrix} C_{\mathrm{g}}(t_0) \\ C_{\mathrm{e}}(t_0) \end{pmatrix}, \qquad (2) \end{aligned}$$

with $\theta = \Omega\delta t$, $\eta = \Delta/\Omega$, $\xi = \Omega_{\mathrm{R}}/\Omega$, and $\Omega = \sqrt{\Omega_{\mathrm{R}}^2 + \Delta^2}$, where Ω_{R} is the Rabi frequency, and $\Delta = \omega - \omega_{\mathrm{l}}$ is the detuning of the laser frequency ω_{l} from the atomic resonance ω. The time $\delta t = t_1 - t_0$ is the duration of the pump excitation, and χ represents the phase of the complex Rabi frequency $\tilde{\Omega}_{\mathrm{R}} = \Omega_{\mathrm{R}}e^{i\chi}$. It is important to note in the formulation given in Eq. (2) that the matrix A, which describes the time evolution of the atomic state during the pump excitation, is given in the lab frame, in which the field-free evolution of the atomic state between the pump and probe excitations is described.

The matrix for the excitation can be expressed in the form

$$A = \begin{pmatrix} |c_g| e^{i\phi/2} & i|c_e| e^{i\zeta/2} \\ i|c_e| e^{-i\zeta/2} & |c_g| e^{-i\phi/2} \end{pmatrix}, \quad (3)$$

where ϕ and ζ are phases of the complex amplitudes c_g and c_e, respectively. The pump and probe excitation operation on the ground state $|g\rangle$ results in

$$\begin{aligned} |\psi(\tau)\rangle &= A \exp(-iH\tau/\hbar) A |g\rangle \quad (4) \\ &= \begin{pmatrix} |c_g|^2 e^{i\phi} e^{i\frac{\omega}{2}\tau} - |c_e|^2 e^{-i\frac{\omega}{2}\tau} \\ i|c_g||c_e| e^{-i\zeta/2} (e^{i\phi/2} e^{i\frac{\omega}{2}\tau} + e^{-i\phi/2} e^{-i\frac{\omega}{2}\tau}) \end{pmatrix} \end{aligned}$$

Therefore, the population in the excited state $P(\tau) = \langle \psi(\tau)|e\rangle\langle e|\psi(\tau)\rangle$ that remains after the probe excitation is given by

$$P(\tau) = 2|c_g|^2 |c_e|^2 (1 + \cos(\omega\tau + \phi)). \quad (5)$$

Here only the phase ϕ remains in the Ramsey signal, while the phase ζ is cancelled out. According to Eq. (2) and Eq. (3) the phase ϕ is defined by

$$\tan(\phi/2) = \frac{\Omega \sin(\omega_1 \delta t/2) \cos(\Omega \delta t/2) - \Delta \cos(\omega_1 \delta t/2) \sin(\Omega \delta t/2)}{\Omega \cos(\omega_1 \delta t/2) \cos(\Omega \delta t/2) + \Delta \sin(\omega_1 \delta t/2) \sin(\Omega \delta t/2)}. \quad (6)$$

When the laser detuning vanishes ($\Delta = 0$) we obtain $\phi = \omega_1 \delta t = \omega \delta t$. Similarly, if $\Omega_R \rightarrow 0$, the phase ϕ converges to $\omega \delta t$. In the regime with $|\Delta| >> |\Omega_R|$ where $\Omega = \sqrt{\Omega_R^2 + \Delta^2} \approx \Delta + \frac{\Omega_R^2}{2\Delta}$, the effects of the laser field can be characterized by the AC-Stark shift $\omega_{ac} = \frac{\Omega_R^2}{4\Delta}$, so that we obtain

$$\tan(\phi/2) \approx \tan\left(\left(\omega - \frac{\Omega_R^2}{2\Delta}\right) \delta t/2\right). \quad (7)$$

Therefore the phase aquired during the excitation is given by the AC-Stark-shifted phase-evolution

$$\phi \approx (\omega - 2\omega_{ac})\, \delta t. \quad (8)$$

If the interactions among the atoms are neglected during the ps pulses, this intrapulse phase is common to the higher- and lower-density ensembles under the condition that their sizes, shapes,

and positions are identical. Accordingly, the intrapulse phase is cancelled out in the phase-shift between those two ensembles.

In the actual measurements, however, slight differences between the sizes, shapes, and positions of the higher- and lower-density ensembles can lead to different intrapulse phases due to different AC-Stark shifts (see Methods section "Estimation of the atom density" in the main text). Additional intrapulse phases due to the Rydberg interactions during the pump and probe excitations are considered to be negligibly small as seen in the theoretical simulations demonstrated in the next paragraph.

We have performed theoretical simulations of the Ramsey signals to investigate the effects of the Rydberg interactions during the pump (or probe) excitation, whose width is 10 ps, on the contrast and phase of the Ramsey oscillations. We consider a two-level system with its level spacing that corresponds to a frequency of 1.008×10^{15} Hz and is the energy difference between 5S and 42D states. We assume the van der Waals interaction $C_6 \sim 34$ GHz μm^6 as it has been obtained in the beyond-mean-field analyses of our data in the main text. The interatomic distance that gives the interaction timescale comparable to the width of the excitation $\sim$ 10 ps is estimated to be $\sim$ 0.84 μm. The average number of atoms within a sphere whose radius is 0.84 μm is estimated to be less than two in our higher-density ensemble. The number of atoms N that has been considered in the present simulation is five and is thus large enough to investigate the interaction effects within the $\sim$ 10 ps pulse excitation. We have compared the populations of the excited state generated by an effective off-resonant two-photon excitation with and without the atom-atom interactions during the excitation process. We have considered the atoms to be randomly distributed within a cube with a volume $r_0^3 = N/n_{\text{av}}$ where the average atom density $n_{\text{av}} = (1.3/2^{(3/2)})\times 10^{12}$ cm^3 is the same value as the one in our experiment. We plot the excited state populations at the end of the excitation pulse as functions of the pulse duration with and without the atom-atom interactions in Supplementary Fig. 7a. Here the red and blue curves show the results with and without the interactions, respectively, during the excitation. We fixed the average Rabi frequency to 18.88 GHz, and the detuning from resonance emerging due to the two-photon light shift to 150 GHz, so that the resulting populations are $\sim$ 3 % in

the excited state at the pulse duration of 10 ps. It is seen in Supplementary Fig. 7a that the populations are almost the same between these two cases with their population difference being less than 1 % at 10 ps, indicating that the interaction effects on the excited-state population are almost negligible during the pulse excitation. Next we have simulated the Ramsey signals with the pump and probe excitations and evaluated the intrapulse-interaction effects on the contrast and phase of the Ramsey oscillations. The simulated Ramsey oscillations are shown in Supplementary Fig. 7b where the red and blue curves show the results with and without the interactions during the excitation pulses, respectively, around the pump-probe delays τ = 400 ps. We calculate the contrasts for those traces and obtain 0.867 and 0.844 with and without the interactions, respectively. The difference between the contrasts is thus $\sim$ 3 %, originating from the interaction during the the excitations. The phase difference between those two traces is less than $\sim$ 1 degrees. This corrsponds to the intrapulse phase discussed in the preceding paragraph. These simulated values for the contrast reduction and the phase difference are much smaller that the measured contrast-decay and the amount of phase-shift seen in Figs. 3 and 6. It is thus concluded that the interaction effects on the contrast and phase are almost negligible. It is also concluded that the contribution of the interactions to the intrapulse delay is negligibly small.

We have verified that the discussion above holds entirely also for a three-level system with rectangular laser pulses. We consider a system consisting of a ground state $|g\rangle$, an intermediate state $|i\rangle$, and a Rydberg excited state $|e\rangle$. A laser field with frequency ω_{l1} couples the state $|g\rangle$ to the upper state $|i\rangle$ with Rabi frequency Ω_{R1} and detuning $\Delta_1 \equiv \omega_{l1} - \omega_1$, where ω_1 is the corresponding transition frequency. A second laser field with Rabi frequency Ω_{R2} drives the transition between the states $|i\rangle$ and $|e\rangle$ with the transition frequency ω_2. In the case that $|\Delta_1| \gg |\Omega_{R1}|, |\Omega_{R2}|$, we can neglect the excitation to the intermediate state, so that the three-level system is effectively reduced to a two-level system. In our actual experiments, however, we use an excitation laser-pulse whose envelop is not rectangular. The condition $|\Delta_1| \gg |\Omega_{R1}|, |\Omega_{R2}|$ holds in the beginning and end of the pulse, whereas it might be violated at its peak intensity around the middle of the pulse, so that the intermediate state $|i\rangle$ can be populated at the

peak intensity. However, this state $|i\rangle$, which is not a Rydberg state, has a negligibly small intrapulse-interaction effect during the excitation pulse and due to adiabaticity given by the condition $\omega_1, (\omega_2 - \omega_1) \gg |\Omega_{R1}|, |\Omega_{R2}|$ no population remains in the intermediate state $|i\rangle$ after the pulse. This shows that our two-photon excitation can be effectively treated as a one-photon excitation. The discussions above on the intrapulse phase and the interaction effects during the excitation also apply to the two-photon excitation in our current experiments.

Supplementary Note 3: Field ionization spectra and estimation of excitation bandwidth

In order to reduce the number of Rydberg states to be excited, the spectra of the ps IR and blue pulsed lasers were cut to be about 0.13 nm and 0.20 nm, respectively, with pulse shapers in a 4f configuration ($f = 500\,\mathrm{mm}$). These bandwidths were common to the Ramsey measurements of the three different Rydberg states $\nu = 38, 42$, and 50. Supplementary Figure 4 shows state-resolved field-ionization spectra measured by ramping the electric field slowly on the microsecond timescale. The spectra indicate that a single state was predominantly populated for each of the excitations to $\nu = 38$ and 42 as seen in Supplementary Figs. 4a and b, whereas more levels were populated for the excitation to $\nu = 50$ as seen in Supplementary Fig. 4c. This is because the energy levels of Rydberg states are inversely dependent on ν^2 and more congested for higher states. Due to the characteristics of field ionization, the threshold value of the electric field to induce ionization depends on ν^{-4} [2], so that higher states are less resolvable with the same ramp-up speed of the electric field. Therefore the ramp-up speed was slower for $\nu = 50$ than for $\nu = 38$ and 42 by a factor of 2/3.

The bandwidth of our Rydberg excitation with the IR and blue pulses was determined from the field-ionization spectrum for $\nu = 42$ presented in Fig. 1c as well as in Supplementary Figs. 4b. Assuming a symmetrical population distribution with respect to the center level $\nu = 42$, we estimated the relative populations in the excited Rydberg states to be $\sim 84\,\%$ for the center state 42D and $\sim 8\,\%$ for each neighboring state 43D and 41D, respectively, from the integrated areas of the ion signal peaks. In Supplementary Figs. 4b, the integration range for

the 43D state (the 42D and 41D states) is indicated by the red-shaded (blue-shaded) region. We did not deconvolute the ion spectrum for those integrations although those peaks were not fully resolved. Based on the relative populations and assuming a Gaussian excitation spectrum, we obtained a bandwidth of ~150 GHz (FWHM).

Supplementary Note 4: Structures in the pump-probe delay dependence of the Ramsey signal

We have made another set of supplementary Ramsey measurements with smaller steps of the pump-probe delay τ than those of the measurements shown in the main text. The results of those supplementary measurements are shown in Supplementary Fig. 8a, in which the red and blue dots show the contrasts of the Ramsey oscillation for the higher- and lower-density ensembles, respectively, as functions of τ. It should be noted here that Supplementary Fig. 8b shows a field ionization spectrum associated with the Ramsey measurement shown in Supplementary Fig. 8a, indicating the 41D, 42D, and 43D states predominantly populated, similar to the measurements shown in the main text, but more fractions of the 41D and 43D states included in these supplementary measurements with a broader bandwidth of the ps excitation laser pulse. It is thus reasonable that the recurrence motion of the Rydberg wave-packet with a period of $\sim$ 10 ps is more pronounced and better resolved in Supplementary Fig. 8a than in Fig. 2d in the main text due to the more fractions of the neighboring levels and smaller steps of τ, respectively. The red- and blue-shaded parts shown in Supplementary Fig. 8a correspond to the recurrence motions of the Rydberg wave-packet numerically simulated only with those three Rydberg levels and with the decay factor exp($-\alpha\sqrt{\tau}$) seen in Eq. (5) in the main text. It is seen from this figure that the simulated results show excellent agreements with the measured τ dependences of the contrast, demonstrating that our Ramsey signal is not affected by other angular momentum states such as S, P, and F states or by the oscillation resulting from the ground-state hyperfine splitting.

It is understood from the comparison between the structures seen at $\tau \sim 130 - 170$ ps in Fig. 2d in the main text and Supplementary Fig. 8a that those oscillations can be assigned to the recurrence motion of the wave-packet. It is also understood from the comparison between

the collapse and revival of the wave packet seen in Supplementary Fig. 8a, which is due to an anharmonicity of the Rydberg levels, and the global structure seen in Fig. 2d that the structures on the ~ 100 ps timescale seen in Fig. 2d are not always assigned to the collapse and revival, but also to experimental fluctuations.

Supplementary Note 5: Active control of many-body dynamics

Supplementary Note 5-1: Atom-density dependence of the Ramsey-contrast decay

Figure 4b in the main text shows the Ramsey contrasts for $\nu = 42$ with a population of 3.5 ± 0.3 % at $\tau = 300$ and 510 ps for several different atom densities ranging from the lower to the higher densities described in the main text. These measurements were made by changing the power of the dipole-trap laser and thereby the trap depth. At first the atom densities in these Ramsey measurements were estimated solely from the total number of atoms and the size of the atomic ensemble obtained by in-situ absorption imaging with a CCD camera without expanding the atomic ensemble, but were underestimated because of the spatial resolution, as is described in Methods section "Estimation of the atom density" in the main text. Therefore it was calibrated in a later independent experiment in which the trapping conditions (1) and (3) (the loading sequence and the dipole-trap laser focusing), which were described in Methods section "Estimation of the atom density", were almost the same as those employed in the Ramsey measurements. In this calibration experiment, we measured the radial trap-frequency, the temperature, the axial size, and the total number of atoms to obtain the atom density as a function of the power of the dipole-trap laser. Supplementary Figure 9 shows the results of this calibration measurement accompanied by a calibration curve, which is a linear interpolation function. The atom densities in the Ramsey measurements were estimated from this calibration curve as a function of the dipole-trap-laser power as shown by the blue diamond-shaped data points in Supplementary Fig. 9, and then the Ramsey contrasts measured at τ =300 and 510 ps are plotted against these estimated atom densities in Fig. 4b in the main text. It is seen in Fig. 4b that the contrast decay is accelerated as the atom density is increased.

Supplementary Note 5-2: The principal-quantum-number dependences of the phase-shift

Figure 4a in the main text shows the Ramsey contrasts as functions of τ for three different Rydberg levels $\nu = 38$, 42, and 50. The populations p_e and estimated peak atom-densities are $p_e \sim 3.2\,\%$ and $\sim 1.2 \times 10^{12}\,\mathrm{cm}^{-3}$ for $\nu = 38$, $p_e \sim 3.3\,\%$ and $\sim 1.3 \times 10^{12}\,\mathrm{cm}^{-3}$ for $\nu = 42$, and $p_e \sim 3.1\,\%$ and $\sim 1.2 \times 10^{12}\,\mathrm{cm}^{-3}$ for $\nu = 50$, respectively (see Methods section "Estimation of the atom density" in the main text for these density estimations). It is seen from this figure that the dephasing is accelerated by increasing the principal quantum number ν of the Rydberg level. We have also measured the corresponding phase-shifts for these three levels simultaneously with the Ramsey contrasts as shown in Supplementary Fig. 10. The theory-model simulations with the continuum approximation indicated by solid lines agree well with the measured results. It should be noted that several Rydberg states are excited in the case of $\nu = 50$, whereas we have considered an excitation only to a single Rydberg state to perform the simulations for all of the three Rydberg levels.

Supplementary Note 6: Calculation of the Ramsey contrast and phase-shift with nearest-neighbor interactions

We follow previous Ramsey studies on Rydberg interactions [3, 4] to calculate the Ramsey-contrast-decays and the phase-shifts expected for nearest-neighbor interactions. In the Ramsey measurement in the main text, the population in the Rydberg state is observed as a function of the delay time τ between the pump and probe excitations. Within the delay time, nearest neighbor atoms evolve under a Hamiltonian

$$H = \sum_{j=1}^{2} \left(E_g |g\rangle_j \langle g|_j + E_e |e\rangle_j \langle e|_j \right) + U(r) |e\rangle_1 |e\rangle_2 \langle e|_2 \langle e|_1, \tag{9}$$

where $|g\rangle$ and $|e\rangle$ are ground- and excited Rydberg-states with energies E_g and E_e, respectively, and $U(r)$ is an interaction energy between a pair of Rydberg atoms separated by r. The time-domain Ramsey signal is obtained by solving a Schrödinger equation with this Hamiltonian

as

$$\begin{aligned} P(\tau) &= 2p_{\mathrm{g}}p_{\mathrm{e}}\left(1 + p_{\mathrm{g}}\cos(\omega\tau + \phi) + p_{\mathrm{e}}\cos((\omega + U(r)/\hbar)\tau + \phi)\right) \\ &= 2p_{\mathrm{g}}p_{\mathrm{e}}\Re\left\{1 + e^{i(\omega\tau+\phi)}\left(p_{\mathrm{g}} + p_{\mathrm{e}}e^{\frac{iU(r)\tau}{\hbar}}\right)\right\}. \end{aligned} \tag{10}$$

Here p_{g} and p_{e} are the ground- and Rydberg-state populations, respectively, $\omega = (E_{\mathrm{e}} - E_{\mathrm{g}})/\hbar$ is the atomic-resonance frequency, and ϕ is the phase offset arising from the AC-Stark shifts during the pulse excitation. This result is identical to the Ramsey signal obtained by Eq. (3) in the main text with $N = 2$. In a homogeneous atom distribution with a density of n, the nearest-neighbor distribution is given by

$$Pr(n, r) = \exp\left(-\frac{4\pi n r^3}{3}\right)4\pi n r^2, \tag{11}$$

where $\int_0^\infty Pr(n, r)dr = 1$. The Ramsey signal given by Supplementary Eq. (10) is averaged over this distribution as follows:

$$\begin{aligned} P_{\mathrm{av}}(n, \tau) &= \int_0^\infty Pr(n, r)P(\tau)dr \\ &= 2p_{\mathrm{g}}p_{\mathrm{e}}\Re\left\{1 + e^{i(\omega\tau+\phi)}\left(p_{\mathrm{g}} + p_{\mathrm{e}}\left[\int_0^{r_{\mathrm{B}}} Pr(n, r)dr + \int_{r_{\mathrm{B}}}^\infty Pr(n, r)e^{\frac{iU(r)\tau}{\hbar}}dr\right]\right)\right\}, \end{aligned} \tag{12}$$

where r_{B} is the blockade radius determined by the finite bandwidth of the excitation with the IR and blue pulses.

This is further averaged over the density distribution of our atomic ensemble, which can be modeled by a Gaussian distribution to be

$$n(\mathbf{x}) = n_{\mathrm{p}}e^{-\frac{(x^2+y^2)}{2\sigma_{\mathrm{xy}}^2}-\frac{z^2}{2\sigma_{\mathrm{z}}^2}}, \tag{13}$$

where $n_{\mathrm{p}} = \frac{N}{(2\pi)^{3/2}\sigma_{\mathrm{xy}}^2\sigma_z}$ is the peak density, N is the number of atoms in the ensemble, σ_{xy} is the width in the x,y-direction, and σ_{z} is the width in the z-direction of the atomic ensemble. The Ramsey signal thus averaged over the whole ensemble is given by

$$\begin{aligned} P_{\mathrm{av}}(\tau) &= \frac{4\pi}{N}\int_0^\infty d\rho\rho^2 n(\rho)P_{\mathrm{av}}(n(\rho), \tau) \\ &= \frac{2}{\sqrt{\pi}n_{\mathrm{p}}}\int_0^{n_{\mathrm{p}}} dn\sqrt{\ln\left(\frac{n_{\mathrm{p}}}{n}\right)}P_{\mathrm{av}}(n, \tau), \end{aligned} \tag{14}$$

where the radius ρ is defined by $\rho = \sqrt{x^2 + y^2 + \left(z\frac{\sigma_{xy}}{\sigma_z}\right)^2}$. In going from the first line to the second line in Supplementary Eq. (14), an integral over the volume of the ensemble is converted to an integral over the density.

It should be noted that the maximum threshold of the Ramsey-contrast-decay expected for nearest-neighbor interactions is determined by the population p_e of a Rydberg state as follows. When the Ramsey oscillations are maximally dephased, the third term in the parentheses in the first line of Supplementary Eq. (10) vanishes. In this limit, the oscillation contrast and the phase-shift converge to p_g $(= 1 - p_e)$ and ϕ, respectively, seen in the second term. These features do not depend on the character of the interaction $U(r)$ such as van der Waals and dipole-dipole.

Supplementary Note 7: Mean-field model

For the mean-field approximation we follow the notation $|e\rangle_j\langle e|_j = P_{ee}^{(j)}$ which results in the following N-atom Hamiltonian

$$H = \sum_{j=1}^{N} E_e P_{ee}^{(j)} + \sum_{j=1}^{N-1} \sum_{i>j}^{N} U(r_{i,j}) P_{ee}^{(i)} P_{ee}^{(j)}, \tag{15}$$

where E_e is the energy of Rydberg state and $U(r_{i,j})$ describes the interaction between atoms i and j separated by $r_{i,j}$. Without loss of generality we have set the ground-state energy $E_g = 0$. By using a relation $P_{ee}^{(j)} = \langle P_{ee}^{(j)} \rangle + \delta P_{ee}^{(j)}$ where $\langle P_{ee}^{(j)} \rangle$ and $\delta P_{ee}^{(j)}$ are the mean value and fluctuation of $P_{ee}^{(j)}$, respectively, we obtain the mean-field approximation from

$$\begin{aligned} P_{ee}^{(i)} P_{ee}^{(j)} &\approx \langle P_{ee}^{(i)} \rangle \langle P_{ee}^{(j)} \rangle + \langle P_{ee}^{(i)} \rangle \delta P_{ee}^{(j)} + \langle P_{ee}^{(j)} \rangle \delta P_{ee}^{(i)} \\ &= \langle P_{ee}^{(i)} \rangle P_{ee}^{(j)} + \langle P_{ee}^{(j)} \rangle P_{ee}^{(i)} - \langle P_{ee}^{(i)} \rangle \langle P_{ee}^{(j)} \rangle, \end{aligned} \tag{16}$$

which allows to write the Hamiltonian in the following form

$$\begin{aligned} H &= \sum_{j=1}^{N} H_j \\ &= \sum_{j=1}^{N} \hbar \left(\omega + \left(\langle P_{ee} \rangle - \frac{\langle P_{ee} \rangle^2}{2} \right) \Delta\omega_j \right) P_{ee}^{(j)} - \hbar \left(\frac{\langle P_{ee} \rangle^2}{2} \Delta\omega_j \right) P_{gg}^{(j)}, \end{aligned} \tag{17}$$

where $\omega = E_e/\hbar$ is the atomic-resonance frequency and $\langle P_{ee}^{(j)}\rangle$ is assumed to be common to all the atoms and set to $\langle P_{ee}\rangle$. Each atom can be considered separately, and the interactions enter as shifts of the energy levels with $\hbar\left(\langle P_{ee}\rangle - \frac{\langle P_{ee}\rangle^2}{2}\right)\Delta\omega_j$ and $\hbar\left(\frac{\langle P_{ee}\rangle^2}{2}\Delta\omega_j\right)$ for the Rydberg and ground states, respectively. Here $\Delta\omega_j = \sum_{j\neq i} U(r_{i,j})/\hbar$ is the sum over all interactions with atom 'j'.

In this model, therefore, Rydberg interactions modify only the period of the Ramsey oscillation of each atom as follows

$$P_{\mathrm{mf};j}(\tau) = 2p_g p_e\left(1 + \cos((\omega + p_e\Delta\omega_j)\tau + \phi)\right), \tag{18}$$

where τ is the pump-probe delay, p_g and p_e are the ground- and Rydberg-state populations, respectively, $\langle P_{ee}\rangle = p_e$, and ϕ is the phase offset arising from the AC-Stark shifts during the pulse excitation. Oscillations with slightly different periods are then averaged over the atom distribution. The interferogram obtained in the Ramsey measurement is thus given by

$$P_{\mathrm{mf}}(\tau) = \frac{1}{N}\sum_{j=1}^{N} P_{\mathrm{mf};j}(\tau) = 2p_g p_e\left(1 + \frac{1}{N}\sum_{j=1}^{N}\cos((\omega + p_e\Delta\omega_j)\tau + \phi)\right). \tag{19}$$

This averaging yields a contrast decay of the Ramsey oscillation. By employing the Monte Carlo method to model a realistic distribution of the N atoms as given by Supplementary Eq. (13) we acquire the results of Supplementary Eq. (19).

Supplementary Note 8: Outline of the least-squares fitting

The least-squares fitting for Fig. 3b in the main text and Supplementary Figs. 2b and 3b was performed so that the residual between the Ramsey contrasts measured for ~3.3 % population and their simulations was minimum, with reasonable steps of fitting parameters, for 500 atoms, whose configuration was generated by the Monte-Carlo simulation. For each of these 500 atoms, the mean-field energy shift is calculated by considering the interactions with $\sim 6\times10^5$ atoms, and these shifts are averaged over the 500 atoms. The fitting parameters such as the C_6 coefficient obtained in that least-squares fitting were used to calculate the simulated curves shown in Fig. 3 and Supplementary Figs. 2 and 3 with an averaging over 20,000 atoms from the ensemble.

The least-squares fitting for Fig. 6b in the main text and Supplementary Figs. 11b and 12b was also performed so that the residual between the Ramsey contrasts measured for $\sim 3.3\,\%$ population and their simulations with Eq (4) in the main text was minimum with reasonable steps of fitting parameters. The fitting parameters such as the C_6 coefficient obtained in that least-squares fitting were used to calculate the simulated curves shown in Fig. 6 and Supplementary Figs. 11 and 12.

Supplementary Note 9: Effects of the atom-density distribution on the phase-shifts

We present detailed analyses of the effects of the atom-density distribution on the phase-shifts. Supplementary Figure 13a shows the exact and mean-field calculations of the phase-shift for the 42D Rydberg state with the Gaussian distribution of the atom density. The Gaussian distribution has been taken from the Ramsey experiment shown in the main text. Both in the exact and mean-field calculations, the phase-shift is saturated as the Rydberg population p_e increases, as shown in Supplementary Fig. 13a. As the Rydberg population is increased, the initial slope of the phase-shift becomes larger due to stronger interactions, so the phase-shift converges earlier to a value around -40 degrees, and the disagreement between the exact and mean-field results becomes smaller.

This saturation is due to the Gaussian atom-density distribution of our experimental setup as shown in Supplementary Fig. 13b, in which the phase-shifts calculated with two different density distributions (Gaussian and homogeneous) and two different Rydberg populations are compared within the mean-field model. The Gaussian distribution is the same as in Supplementary Fig. 13a, whereas the homogeneous density has been set to the average density of the Gaussian distribution. It is seen that the Gaussian distribution gives the phase-shift saturated more rapidly for the higher Rydberg population. With the homogeneous distribution, however, the phase-shifts are not saturated within this timescale 1000 ps. In contrast to the homogeneous distribution, the rapid decrease of the atom density in the Gaussian tails results in the saturation of the phase-shift. This is because the contribution to the phase-shift from

atoms distant from the center of the Gaussian distribution is suppressed, and therefore the phase-shift does not grow afterwards. The stronger interactions for $p_\mathrm{e} \sim 3.3\,\%$ yield the phase-shift saturated within our measurement time 500 ps. This saturation is reached both by the exact and mean-field calculations for $p_\mathrm{e} \sim 3.3\,\%$ as seen in Supplementary Fig. 13a, unlike the case with $p_\mathrm{e} \sim 1.2\,\%$ and therefore weaker interactions, giving the closer agreement between the mean-field and exact results as well as the experimental ones for $p_\mathrm{e} \sim 3.3\,\%$ than for $p_\mathrm{e} \sim 1.2\,\%$, as seen in Fig. 3d in the main text.

Supplementary Note 10: Numerical simulations with alternative forms of interactions

Supplementary Note 10-1: Effective treatment of a two-atom interaction

At short interatomic distances a dipole-dipole interaction in non-diagonal terms of the Hamiltonian couples the initial Rydberg states of a pair of atoms described by $|\cdots \mathrm{e} \cdots \mathrm{e} \cdots\rangle$ with other Rydberg states $|\cdots \mathrm{e}' \cdots \mathrm{e}'' \cdots\rangle$. Such couplings induce hybridization among multiple Rydberg states, leading to congested potential structures. To handle this intractable problem in the present study, we have considered a single effective potential for the two-atom interaction that represents the congested potential structure. Thereby, the effective interaction enters only the diagonal components of the Hamiltonian.

Supplementary Note 10-2: Numerical simulation with an anisotropic van der Waals interaction

In the main text we have considered an isotropic van der Waals interaction given by $U(r) = -C_6/r^6$. Here we introduce an anisotropy into the van der Waals interaction by

$$U(r,\theta) = -\frac{C_6(1 - 3\cos^2(\theta))^2}{r^6}, \tag{20}$$

where θ is the angle between the z-axis (Fig. 1a) and a line connecting two atoms interacting with each other. The comparisons between our experimental observations and the simulations with the anisotropic potential $U(r,\theta)$ above are presented in Supplementary Fig. 2 for the mean-field model and in Supplementary Fig. 11 for the theory model beyond mean-field with

the analytical continuum-approximation, respectively. It is seen in Supplementary Fig. 2c that the mean-field simulation fails to reproduce the observed phase-shift again, whereas the theory-model simulations agree with the measured Ramsey contrasts and phase-shifts for both of the Rydberg populations p_e ~1.2 % to 3.3 %. The adjustment parameter employed in these simulations with the anisotropic potential is $C_6 = 63\,\mathrm{GHz}\,\mu\mathrm{m}^6$ and is comparable to $C_6 = 34\,\mathrm{GHz}\,\mu\mathrm{m}^6$ employed in the simulations without the potential anisotropy.

Supplementary Note 10-3: Numerical simulation with a hybrid form of a dipole-dipole and a van der Waals interaction and with a pure dipole-dipole interaction

A hybrid form of a dipole-dipole and a van der Waals (DD-vdW) interaction is defined by

$$U(r) = \begin{cases} -\frac{C_3}{r^3} & r \le r_c \\ -\frac{C_6}{r^6} & r > r_c \end{cases}, \tag{21}$$

where r_c is the crossover radius defined by $r_c^3 = C_6/C_3$. We have performed numerical simulations of the Ramsey contrast and phase-shift using this DD-vdW interaction with C_3 and r_c being fitting parameters.

We have also performed the simulations using a dipole-dipole (DD) interaction without anisotropies. The results of those simulations are presented in Supplementary Fig. 3 for the mean-field model and in Supplementary Fig. 12 for the theory model with the continuum approximation, respectively. The corresponding results with the pure van der Waals (vdW) interaction, which have been shown in Figs. 3 and 6 of the main text, are presented again in Supplementary Figs. 3 and 12 to be compared with the DD-vdW and DD results. It is seen in Supplementary Fig. 3c that the mean-field simulation fails to reproduce the observed phase-shift again for both the DD-vdW and DD interactions. It is seen in Supplementary Fig. 12, on the other hand, that the theory model simulations beyond mean-field with the DD-vdW interaction show good agreements simultaneously with the Ramsey contrasts and phase-shifts, whereas similar agreements have not been found with the DD interaction. Supplementary Figure 1 shows the two-atom potentials that we have obtained by diagonalizing the ^{87}Rb Hamiltonian for two

atoms with the DD interaction. It is seen from this figure that there is a mixing of states correlating to the $42D_{5/2}$+$42D_{5/2}$ and $44P_{3/2}$+$40F_{7/2}$ asymptotes due to DD coupling at interatomic distances shorter than $\sim$ 2 µm. This distance is longer than the shortest interatomic distance accessible by our ps pulse excitation, indicating the validity of the DD-vdW interaction. The DD-vdW interaction, however, needs two fitting parameters C_3 and r_c, whereas the pure vdW interaction needs only one fitting parameter C_6. We have, therefore, employed the pure vdW interaction in the main text to suppress the ambiguity of the fitting.

Supplementary Figure 5 shows the simulated Ramsey contrast and phase-shift at $\tau = 500$ ps as functions of the cutoff radius r_0 (the lower abscissa), at which we truncate the integration in Eq. (6) in the main text, and of an average number of interacting atoms within the volume $V = \frac{4\pi}{3}(r_0^3 - r_B^3)$ (the higher abscissa). They are simulated by the theory model with the continuum approximation for the vdW, DD, and DD-vdW interactions without anisotropies. The population of the $42D_{5/2}$ is set to ~3.3% in these simulations. The converged values agree well with the measured ones indicated by the dark-grey solid lines, each of which is the average over eight points around $\tau = 500$ ps in Figs. 6b or d in the main text. The light-grey shaded area represents one standard deviation of the average over those eight measured values. On the other hand, the Ramsey contrast and phase-shift simulated with the DD interaction neither converge nor agree with the experimental observations. The black solid line in Supplementary Fig. 5a shows the Ramsey contrast $|g(\tau)|$ given by Eq. (7) in the main text with $\gamma(\tau) = 0$, which gives the situation in which the Ramsey oscillations modulated by the Rydberg-Rydberg interactions are dephased completely. This black solid line thus represents the upper limit of the contrast decay and accordingly the lower limit of the number of atoms $\sim$ 32 to reproduce the Ramsey contrast $\sim$ 0.45 measured at $\tau = 500$ ps, irrespective of the potential curves.

Supplementary References

[1] Tanner, P. J., Han, J., Shuman, E. S. & Gallagher, T. F. Many-body ionization in a frozen Rydberg gas. *Phys. Rev. Lett.* **100**, 043002 (2008).

[2] Gallagher, T. F. *Rydberg Atoms.* (Cambridge University Press, New York, 2005).

[3] Nipper, J. *et al.* Atomic pair-state interferometer: controlling and measuring an interaction-induced phase shift in Rydberg-atom pairs. *Phys. Rev. X* **2**, 031011 (2012).

[4] Zhou, T., Li, S. & Jones, R. R. Rydberg-wave-packet evolution in a frozen gas of dipole-dipole-coupled atoms. *Phys. Rev. A* **89**, 063413 (2014).
